# GeoSentinel Analysis of Travelers’ Diarrhea Antimicrobial Resistance Patterns

**DOI:** 10.1001/jamanetworkopen.2025.51089

**Published:** 2025-12-22

**Authors:** Bhawana Amatya, Prativa Pandey, Sarah L. McGuinness, Martin P. Grobusch, Stephen Muhi, Karin Leder, Marta Díaz-Menéndez, Giacomo Stroffolini, Federico Gobbi, Inés Oliveira-Souto, Jesse J. Waggoner, Caroline Theunissen, Rosa de Miguel, Kescha Kazmi, Suvash Dawadi, Rashila Pradhan, Abraham Goorhuis, Bradley A. Connor, Davidson H. Hamer, Daniel T. Leung

**Affiliations:** 1 CIWEC Hospital and Travel Medicine Center, Lainchaur, Kathmandu, Nepal; 2 School of Public Health and Preventive Medicine, Monash University, Melbourne, Australia; 3 Department of Infectious Diseases, Alfred Health, Melbourne, Australia; 4 Center of Tropical Medicine and Travel Medicine, Department of Infectious Diseases, Amsterdam University Medical Centers, Location AMC, University of Amsterdam, Amsterdam, the Netherlands; 5 Department of Microbiology and Immunology, Peter Doherty Institute for Infection and Immunity, The University of Melbourne, Melbourne, Victoria, Australia; 6 Victorian Infectious Diseases Service, Royal Melbourne Hospital, Parkville, Victoria, Australia; 7 Tropical Medicine Department, Hospital Universitario La Paz-Carlos III, IdIPAz, and CIBERINFECT, Madrid, Spain; 8 Department of Infectious-Tropical Diseases and Microbiology, IRCCS Sacro Cuore Don Calabria Hospital, Negrar di Valpolicella, Verona, Italy; 9 Division of Infectious Diseases, Department of Medicine, Lausanne University Hospital (CHUV), Lausanne, Switzerland; 10 Department of Clinical and Experimental Sciences, University of Brescia, 25121 Brescia, Italy; 11 International Health Unit Vall d’Hebron-Drassanes, Infectious Diseases Department, Vall d’Hebron University Hospital, PROSICS Barcelona, Barcelona, Spain; 12 Centro de Investigacion Biomédica en Red de Enfermedades Infecciosas (CIBERINFEC), Instituto de Salud Carlos III, Madrid, Spain; 13 Division of Infectious Diseases, Emory University Department of Medicine, Atlanta, Georgia; 14 Department of Clinical Sciences, Institute of Tropical Medicine, Antwerp, Belgium; 15 Tropical Medicine Department, Hospital Universitario La Paz-Carlos III, IdIPAz, and CIBERINFECT, Madrid, Spain; 16 Division of Infectious Diseases, Department of Pediatrics, The Hospital for Sick Children, University of Toronto, Toronto, Canada; 17 Weill Cornell Medical College and the New York Center for Travel and Tropical Medicine, New York, New York; 18 Department of Global Health, Boston University School of Public Health, Boston, Massachusetts; 19 Section of Infectious Diseases, Department of Medicine, Boston University Chobanian & Avedisian School of Medicine, Boston, Massachusetts; 20 Center on Emerging Infectious Diseases, Boston University, Boston, Massachusetts; 21 Division of Infectious Diseases, Department of Internal Medicine, University of Utah School of Medicine, Salt Lake City; 22 Division of Microbiology and Immunology, Department of Pathology, University of Utah School of Medicine, Salt Lake City

## Abstract

**Importance:**

Diarrheal disease causes substantial morbidity in international travelers. Knowledge of antibiotic resistance of causative pathogens helps guide empiric treatment decisions.

**Objective:**

To characterize antimicrobial nonsusceptibility patterns of culture-confirmed *Campylobacter* species, nontyphoidal *Salmonella* (NTS) species, *Shigella* species, and diarrheagenic *Escherichia coli* isolated from international travelers with diarrhea identified by the GeoSentinel Surveillance Network.

**Design, Setting, and Participants:**

This is a retrospective cross-sectional analysis of antimicrobial susceptibility data for 4 major pathogens causing travel-associated diarrhea, reported from April 14, 2015, to December 19, 2022, at 58 of 71 international GeoSentinel sites. Participants included a convenience sample of international travelers with acute diarrhea seen during or after travel presenting at the GeoSentinel sites.

**Main Outcomes and Measures:**

The main outcomes were demographics, clinical characteristics, and antimicrobial susceptibility profiles of patients with culture-confirmed *Campylobacter* species, NTS species, *Shigella* species, and diarrheagenic *E coli* species. Routinely collected antimicrobial susceptibility data with intermediate susceptibility and resistant were defined as nonsusceptible. The antimicrobial susceptibility test results were described as numbers and percentages, and binomial 95% CIs were calculated for the proportions.

**Results:**

Of 859 total cases, the median (IQR) age was 30 (23-43) years, and 440 travelers (51%) were male. Among *Campylobacter* isolates, nonsusceptibility to fluoroquinolones was found in 206 of 274 isolates (75%; 95% CI, 70%-80%), and nonsusceptibility to macrolides was found in 30 of 255 isolates (12%; 95% CI, 8%-16%) and was highest in travelers to South Central Asia (45 of 51 isolates; 88%; 95% CI, 76%-96%). Among NTS species, 96 of 302 isolates (32%; 95% CI, 27%-37%) were nonsusceptible to fluoroquinolones, 18 of 111 isolates (16%; 95% CI, 10%-24%) were nonsusceptible to macrolides, and 15 of 273 isolates (5%; 95% CI, 3%-9%) were nonsusceptible to third-generation cephalosporins. For *Shigella* species, 44 of 196 isolates (22%; 95% CI, 17%-29%) were nonsusceptible to fluoroquinolones, and 36 of 103 isolates (35%; 95% CI, 26%-45%) were nonsusceptible to macrolides. For *E coli*, fluoroquinolone nonsusceptibility was 18% (12 of 66 isolates; 95% CI, 10%-30%). Of note, 19 of 24 *Shigella* isolates (79%; 95% CI, 58%-93%) were nonsusceptible to fluoroquinolones among travelers to South Central Asia, and 29 of 37 *Shigella* isolates (78%; 95% CI, 62%-90%) were nonsusceptible to macrolides among travelers to South America.

**Conclusions and Relevance:**

In this cross-sectional study of travelers’ diarrhea antimicrobial resistance patterns, there was marked variability of nonsusceptibility to 2 major classes of antibiotics commonly used for treating travelers’ diarrhea among global regions. Antimicrobial susceptibility from culture should be obtained when possible, including after pathogen detection by culture-independent methods. These findings may help inform strategies for self-treatment and clinician management of travelers’ diarrhea.

## Key Points

**Question:**

What are the antibiotic nonsusceptibility patterns in travelers’ diarrhea at cases presenting at GeoSentinel sites?

**Findings:**

In this cross-sectional study of 859 cases, nonsusceptibility to fluoroquinolones was 75% for *Campylobacter* species, 32% for nontyphoidal *Salmonella* species, 22% for *Shigella* species, and 18% for diarrheagenic *Escherichia coli* species. Nonsusceptibility to macrolides was 12% for *Campylobacter *species, 16% for nontyphoidal *Salmonella *species, and 35% for *Shigella *species.

**Meaning:**

These findings suggest that there are wide geographic differences in antibiotic nonsusceptibility patterns in travelers’ diarrhea; antimicrobial susceptibility from culture should be obtained when possible to inform strategies for self-treatment and clinician management of travelers’ diarrhea.

## Teaser

This cross-sectional study examines antimicrobial nonsusceptibility patterns of culture-confirmed *Campylobacter* species, nontyphoidal *Salmonella* species, *Shigella* species, and diarrheagenic *Escherichia coli* isolated from international travelers with diarrhea.

## Introduction

Acute diarrhea affects 20% to 88% of international travelers depending on destination, duration, and season of travel.^1,2,3^ The invasive bacterial pathogens *Campylobacter *species,* Shigella *species, and nontyphoidal *Salmonella* (NTS) species, as well as diarrheagenic strains of *Escherichia coli*, are responsible for the majority of cases of travel-associated bacterial diarrhea.^4,5,6^ Antimicrobial resistance among enteropathogenic bacteria is an emerging concern,^7^ and the World Health Organization’s Global Priority Pathogens List includes fluoroquinolone-resistant *Campylobacter*,* Salmonella*, and *Shigella* species.^8^

Current guidelines for the management of moderate-to-severe acute travelers’ diarrhea (TD) recommend azithromycin, fluoroquinolones, or rifaximin for both clinician-initiated and traveler-initiated empiric treatment, although rifaximin use is limited because it is not effective for invasive pathogens.^9,10,11^ The recommended use of azithromycin, especially for travelers to South and Southeast Asia, is based on the documented high prevalence of resistance to fluoroquinolones among bacterial causes of TD in these regions.^6,12,13^ However, data on resistance rates in other global regions are mostly limited to studies of children residing in low-income and middle-income countries,^6,14^ whereas rates of resistance among isolates infecting international travelers are largely limited to single-location studies.^14^ Knowledge of resistance patterns can inform the selection of empiric antibiotics for the treatment of TD based on region of acquisition.

GeoSentinel is a worldwide network of tropical medicine centers located on 6 continents that submit data on travelers, immigrants, and refugees for disease surveillance.^15^ By using the GeoSentinel database, this report aims to describe the antibiotic resistance patterns of 4 common diarrheagenic bacteria in international travelers acquired from various global regions.

## Methods

The GeoSentinel data collection protocol was reviewed by a human participants advisor at Centers for Disease Control and Prevention’s National Center for Emerging and Zoonotic Infectious Diseases and was determined to be public health surveillance and not human participants research requiring submission to institutional review boards. Additional ethical clearances were obtained by sites as needed by their local regulations and respective institutions. Informed consent was not required. This cross-sectional study was conducted and reported in accordance with the Strengthening the Reporting of Observational Studies in Epidemiology (STROBE) reporting guidelines for cross-sectional studies.

GeoSentinel, a network of 71 specialized travel or tropical medicine sites across 6 continents, monitors travel-related illnesses in patients presenting to their clinics and hospitals. Clinicians collect deidentified surveillance data using standardized diagnostic codes, which are then entered into a centralized database.^15^

This retrospective study examined antibiotic nonsusceptibility patterns and demographics of patients with acute diarrhea, defined as diarrhea lasting less than 2 weeks,^16^ related to international travel. Data were extracted from the GeoSentinel database. The inclusion criteria were patients with diarrhea during or after travel caused by *Campylobacter *species, NTS, *Shigella* species, or diarrheagenic *E coli* isolated from culture, with at least 1 antimicrobial susceptibility test (AST) result available, from April 14, 2015, to December 19, 2022. Exclusion criteria were cases where no region of exposure was available and *E coli* infections not associated with acute diarrhea.

Separate datasets were created for the 4 diarrheagenic bacteria: *Campylobacter* species, NTS, *Shigella* species, and diarrheagenic *E coli*. We obtained data on travelers’ demographics (age, sex, and country of birth, citizenship, or residence), trip details (reporting GeoSentinel site, purpose of travel, travel destination, region of travel, and visit date to the GeoSentinel site), and clinical presentation (diagnosis, organism, specimen, syndrome assigned to the GeoSentinel diagnosis code, clinical setting, and AST results). The AST results for all available antibiotics were extracted. After removal of duplicates, the 4 datasets were combined (eFigure 1 in Supplement 1). Intermediate and resistant AST categories were then pooled into a single category of nonsusceptible in accordance with site-specific and country-specific testing methods and guidelines.

Only the most recent travel date was included in the analysis when calculating trip duration for posttravel patients who had multiple travel dates to the same or different country. In cases where further details were needed, the sites were contacted for further information. Extensively drug-resistant (XDR) *Campylobacter* species were defined in our report as isolates that were nonsusceptible to at least 5 of the antibiotic classes. XDR *Shigella* species were defined as *Shigella* species that was resistant to azithromycin, ciprofloxacin, ceftriaxone, trimethoprim-sulfamethoxazole, and ampicillin.^17^

Of the 25 of 58 GeoSentinel sites with AST methods available, 17 used disk diffusion, minimum inhibitory concentrations, and automated systems. Eight sites used a combination of disk diffusion, minimum inhibitory concentration, or completely automated systems. For breakpoint criteria, 13 sites used Clinical and Laboratory Standards Institute, 8 used European Committee on Antimicrobial Susceptibility Testing, 3 used both, and 1 used Antimicrobials Committee of the French Society of Microbiology (eTable 1 in Supplement 1). Most sites used either Clinical and Laboratory Standards Institute–recommended or European Committee on Antimicrobial Susceptibility Testing–recommended American Type Culture Collection strains of bacteria for external and internal quality control and had periodic quality control testing. Discrepancies in testing methods across sites were handled by individual sites using standardized quality control techniques.

### Statistical Analysis

Data analysis and data visualization were performed using R statistical software version 4.3.1 (R Project for Statistical Computing). Quantitative variables were assessed by median, IQR, and range, and 95% CIs were calculated. The AST results were described as numbers and percentages, and binomial 95% CIs were calculated for the proportions. Comparison of tested vs untested isolates is shown in eTable 2 in Supplement 1.

## Results

During the study period, there were 859 cases of bacterial-attributed diarrhea with AST data and region of exposure available from 58 GeoSentinel sites, with median (IQR) age of 30 (23-43) years; 440 travelers (51%) were male, and 419 travelers (49%) were female (eFigures 2, 3, and 4 in Supplement 1). These included 286 cases of *Campylobacter species*, 305 NTS species, 215 *Shigella* species, and 75 diarrheagenic *E coli* (Table). Twenty-one patients (2%) had multiple pathogens identified.

**Table.** Demographics and Clinical Details of Acute Diarrhea Cases in the GeoSentinel Database Attributed to Diarrheagenic Bacteria With Antibiotic Susceptibility Data

Characteristic	Travelers, No. (%)^a^				
	Total (N = 859 [100%])	*Campylobacter* species (n = 286 [33%])	NTS (n = 305 [36%])	*Shigella* species (n = 215 [25%])	Diarrheagenic *E coli* (n = 75 [9%])
Age, median (IQR) [range], y^b^	30 (23-43) [0-82]	27 (21-37) [1-78]	32 (23-44) [0-82]	33 (26-43) [1-80]	31 (24-43) [1-80]
Sex					
Female	419 (49)	139 (49)	131 (43)	117 (54)	43 (57)
Male	440 (51)	147 (51)	174 (57)	98 (46)	32 (43)
Documented pretravel health advice^c^	253 (38)	101 (45)	86 (39)	66 (40)	5 (8)
Reason for travel^d^					
Tourism	513 (62)	169 (61)	190 (65)	115 (57)	48 (69)
VFR (traditional definition)^e^	88 (11)	17 (6)	46 (16)	23 (11)	7 (10)
Business or occupational	65 (8)	22 (8)	18 (6)	20 (10)	5 (7)
Missionary, humanitarian, volunteer, or community service	46 (6)	21 (8)	12 (4)	12 (6)	1 (1)
Corporate or professional	35 (4)	14 (5)	6 (2)	14 (7)	3 (4)
VFR (nontraditional definition)^e^	18 (2)	5 (2)	8 (3)	6 (3)	NA
Conference	12 (1)	5 (2)	1 (<1)	5 (2)	2 (3)
Education or student	12 (1)	7 (3)	4 (1)	1 (<1)	NA
Migration	12 (1)	2 (1)	6 (2)	NA	4 (6)
Research	6 (1)	1 (<1)	1 (<1)	5 (2)	NA
Study abroad	6 (1)	6 (2)	NA	NA	NA
Student	5 (1)	5 (2)	NA	NA	NA
Providing medical care	3 (<1)	1 (<1)	1 (<1)	1 (<1)	NA
Retirement	1 (<1)	NA	1 (<1)	NA	NA
Type of care					
Inpatient intensive care unit	3 (<1)^f^	1 (<1)	3 (<1)	NA	NA
Inpatient ward	199 (23)	33 (12)	126 (41)	35 (16)	12 (16)
Outpatient	657 (77)	252 (88)	176 (58)	180 (84)	62 (84)
Region of exposure					
Sub-Saharan Africa	217 (25)	58 (20)	81 (27)	73 (34)	7 (9)
Southeast Asia	205 (24)	95 (33)	88 (29)	18 (8)	6 (8)
South America	145 (17)	22 (8)	30 (10)	51 (24)	45 (61)
South Central Asia	106 (12)	53 (19)	24 (8)	29 (13)	3 (4)
Caribbean	46 (5)	7 (2)	26 (9)	13 (6)	2 (3)
Central America	43 (5)	14 (5)	16 (5)	12 (6)	3 (4)
North Africa	30 (3)	5 (2)	13 (4)	11 (5)	3 (4)
North East Asia	20 (2)	14 (5)	6 (2)	NA	2 (3)
Middle East	18 (2)	5 (2)	9 (3)	3 (1)	2 (3)
Western Europe	16 (2)	7 (2)	7 (2)	3 (1)	1 (1)
Eastern Europe	5 (1)	4 (1)	1 (<1)	NA	NA
North America	4 (<1)	1 (<1)	1 (<1)	2 (1)	NA
Oceania	3 (<1)	NA	3 (1)	NA	NA
Australia or New Zealand	1 (<1)	1 (<1)	NA	NA	NA
Clinical setting					
Seen after travel	743 (86)	276 (97)	278 (91)	179 (83)	27 (36)
Seen during travel	115 (13)	10 (3)	26 (9)	36 (17)	47 (64)
Migration travel only	1 (<1)	NA	1 (<1)	NA	NA
Onset to visit, median (IQR), d	5 (2-11)	4 (2-9)	6 (3-21)	4 (2-13)	2 (1-7)
Trip duration, median (IQR), d	14 (7-22)	13 (7-21)	15 (8-23)	15 (7-24)	17 (6-30)

Abbreviations: *E coli*, *Escherichia coli*; NA, not applicable; NTS, nontyphoidal *Salmonella* species; VFR, visiting friends and relatives.

^a^ The percentages may not add up to 100 due to codetections. Three patients had codetection of *Campylobacter* and *E coli*. Six had codetection with *Campylobacter* and NTS species. Four had codetection with *Campylobacter* and *Shigella* species. Two had codetection with *E coli* and NTS species. Three had codetection with *E coli* and *Shigella* species, and 3 had codetection with both NTS and *Shigella* species.

^b^ Age was unavailable in 3 cases, 1 each for *Campylobacter *species, NTS, and *E coli.*

^c^ Pretravel encounter data were missing (ie, unknown, not applicable or don’t know) for 197 cases overall, including 62 for *Campylobacter *species, 82 for NTS, 51 for *Shigella *species, and 10 for diarrheagenic *E coli.*

^d^ Reason for travel data was missing for 31 cases overall, and 6 were listed as others, with 9 for *Campylobacter *species, 8 for NTS, 6 for *Shigella *species, and 5 for diarrheagenic *E coli.*

^e^ Traditional VFR means that the individual is traveling from the region in which they are currently residing (usually as a migrant or expatriate or long-term visitor) to their region of origin (a low-income country) to visit friends or relatives. This includes people who are traveling with a child or grandchild (second-generation VFRs) or parent, and those traveling with a spouse or partner. Nontraditional VFR refers to individuals who are traveling to visit friends or relatives but who are not returning to their region of origin or are not traveling to a low-income country.^10^

^f^ One patient admitted to the intensive care unit had both *Campylobacter* species and NTS.

During the study period, GeoSentinel sites reported 3300 cases of acute diarrheal illness where these organisms had been isolated or identified with different diagnostic modalities including culture and polymerase chain reaction (eFigure 5 in Supplement 1). Only a limited fraction had AST data available and many had region of exposure not available.

Isolates with AST data were available for travelers to 103 destination countries, with the top 5 regions of exposure being sub-Saharan Africa (217 travelers [25%]), Southeast Asia (205 travelers [24%]), South America (145 travelers [17%]), South Central Asia (106 travelers [12%]), and the Caribbean (46 travelers [5%]) (eFigure 4 in Supplement 1). The top 3 countries of exposure were Peru, India, and Thailand (eFigure 6 in Supplement 1). The top 3 countries of residence were Spain (112 travelers [14%]), Japan (116 travelers [14%]), and Canada (112 travelers [13%]).

Only 253 of 859 travelers (30%) had received pretravel consultation. The majority were seen after travel (743 travelers [87%]). Tourism was the primary reason for travel for 513 patients (62%) followed by visiting friends and relatives (88 patients [11%]), and business or occupational reasons (65 patients [8%]) (eFigure 7 in Supplement 1); 202 travelers (24%) were treated as inpatients, with 126 travelers (41%) of those infected with NTS treated as inpatients. Three patients (<1%) were admitted to the intensive care unit, with 1 patient having both *Campylobacter* and *Salmonella *species (eFigure 8 in Supplement 1). The median (IQR) time between illness onset and presentation to a GeoSentinel site was 5 (2-11) days. The median (IQR) duration of travel for those presenting to a GeoSentinel site after travel was 14 (7-22) days (Table).

### *Campylobacter* Species

**Figure 1.**
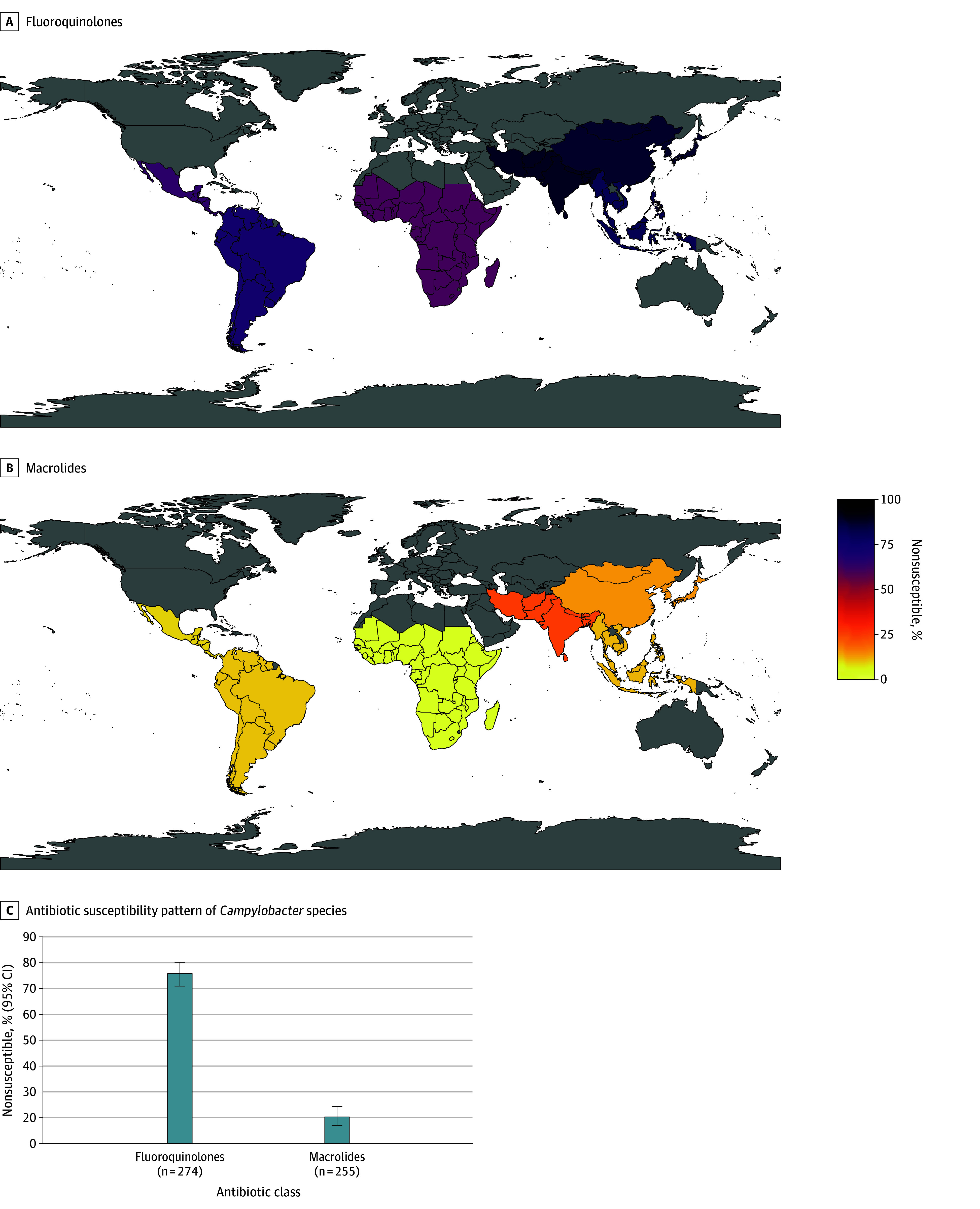
*Campylobacter *Species Nonsusceptibility to Antibiotics by World Region Maps show nonsusceptibility to fluoroquinolones (A) and macrolides (B). Only regions with 10 or more isolates with antimicrobial susceptibility test results are included. Australia and New Zealand (n = 1), Caribbean (n = 6), Eastern Europe (n = 4), Middle East (n = 4), North Africa (n = 5), North America (n = 1), and Western Europe (n = 7) were not included. Panel C shows antibiotic susceptibility pattern of *Campylobacter* species to fluoroquinolones and macrolides (n = 286); not shown are first-generation or second-generation cephalosporins, amoxicillin or ampicillin, β-lactam or β-lactamase inhibitor combinations, chloramphenicol, and cotrimoxazole, which had 100% nonsusceptibility but with data on fewer than 10 isolates.

The predominant regions for acquisition of *Campylobacter* species were Southeast Asia (95 of 286 cases [33%]), sub-Saharan Africa (58 of 286 cases [20%]), and South Central Asia (53 of 286 cases [19%]); the top 3 countries were India (38 cases [14%]), Thailand (29 cases [11%]), and Indonesia (27 cases [10%]) (eFigure 9 in Supplement 1). There were 286 cases of *Campylobacter* with AST data available for at least 1 drug class (eTable 3 in Supplement 1). Most *Campylobacter* species were nonsusceptible to fluoroquinolones (206 of 274 isolates [75%; 95% CI, 70%-80%]) and the highest proportion of nonsusceptibility was seen among travelers to South Central Asia (45 of 51 travelers [88%; 95% CI, 76%-96%]), Southeast Asia (73 of 91 travelers [80%; 95% CI, 71%-88%]), and sub-Saharan Africa (34 of 57 travelers [60%; 95% CI, 46%-72%]) (Figure 1A; eFigure 10 in Supplement 1). Macrolide nonsusceptibility was 12% overall (30 of 255 isolates; 95% CI, 8%-16%) and was most frequently reported from travelers to South Central Asia (12 of 49 isolates [24%; 95% CI, 13%-39%]) and Southeast Asia (9 of 82 isolates [11%; 95% CI, 5%-20%]) (Figure 1B; eFigure 11 in Supplement 1).

There were 26 cases of MDR *Campylobacter* species, which were nonsusceptible to both fluoroquinolones and macrolides. Two cases of XDR *Campylobacter* species were noted in 2016, both outpatients. The first was a 17-year-old boy with a travel history to Malaysia (also nonsusceptible to chloramphenicol), and the second was a 23-year-old woman with a travel history to Singapore.

### NTS Species

**Figure 2.**
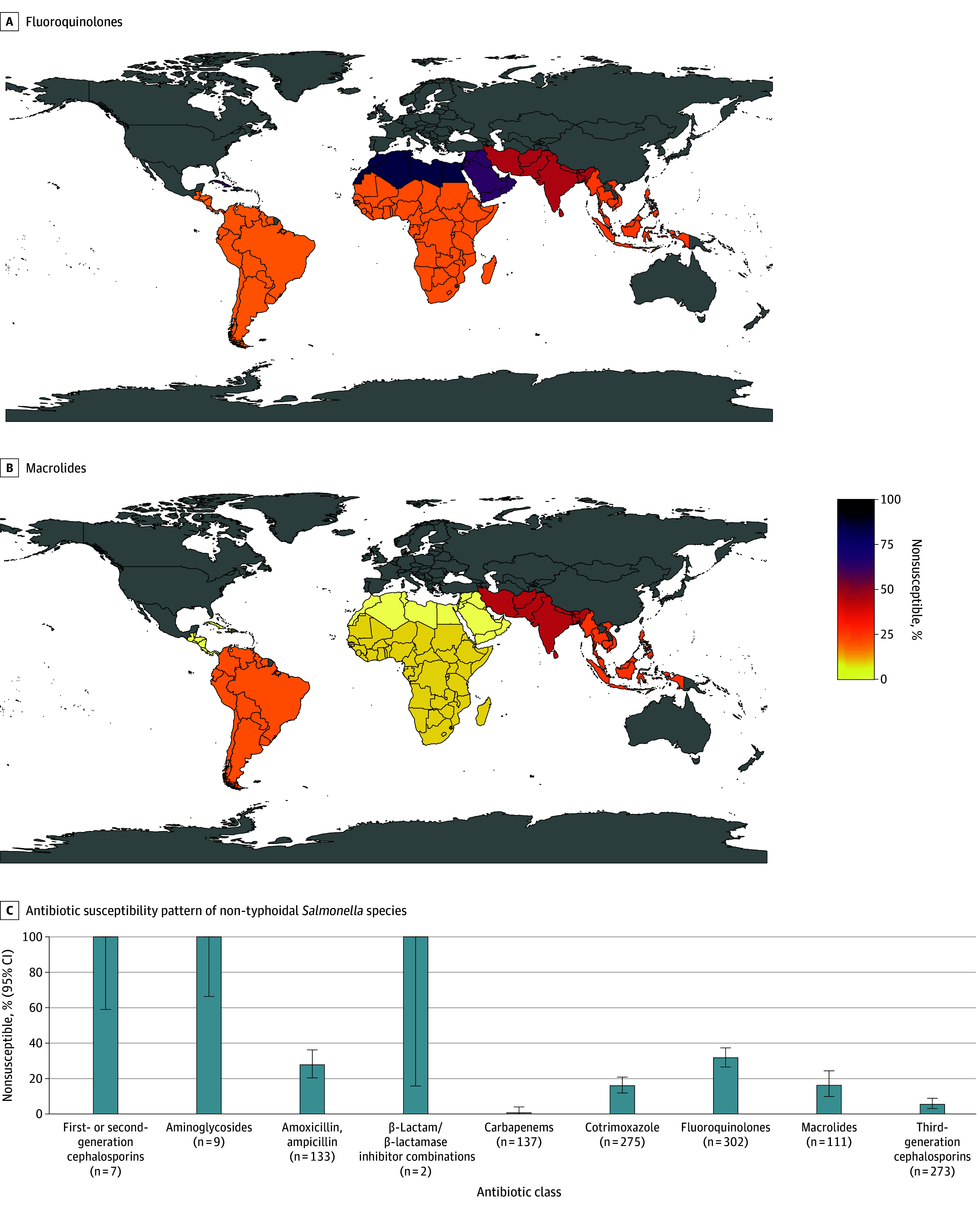
Nontyphoidal *Salmonella* Species Nonsusceptibility to Antibiotics by World Region Maps show nonsusceptibility to fluoroquinolones (A) and macrolides (B). Only regions with 10 or more isolates with antimicrobial susceptibility test results are included. North East Asia (n = 6), Western Europe (n = 7), Eastern Europe (n = 1), North America (n = 1), and Oceania (n = 4) are not included. Panel C shows antibiotic susceptibility pattern of nontyphoidal *Salmonella* species to different antibiotics (n = 305).

There were 305 NTS species cases from a total of 859 travelers with acute diarrhea with AST data (Table). NTS species isolates were predominantly reported from travelers to Southeast Asia (88 travelers [29%]) and sub-Saharan Africa (81 travelers [27%]). The 3 countries with the most reports of NTS species acquisition were Thailand, Indonesia, and Vietnam (eFigure 9 in Supplement 1). Among NTS isolates, 96 of 302 (32%; 95% CI, 27%-37%) were nonsusceptible to fluoroquinolones, 18 of 111 (16%; 95% CI, 10%-24%) were nonsusceptible to macrolides, and 15 of 273 (5%; 95% CI, 3%-9%) were nonsusceptible to third-generation cephalosporins (Figure 2C; eTable 4 in Supplement 1). Rates of nonsusceptibility to fluoroquinolones were 24% (19 of 80 isolates; 95% CI, 15%-35%) among isolates from travelers to Southeast Asia, 45% (10 of 22 isolates; 95% CI, 24%-68%) for South Central Asia, and 62% (18 of 29 isolates; 95% CI, 42%-79%) for the Caribbean (Figure 2A; eFigure 12 in Supplement 1). Among isolates with macrolide AST results (102 of 305 isolates [33%]), the highest proportion of nonsusceptibility was among travelers to South Central Asia (4 of 9 travelers [44%; 95% CI, 14%-79%]) (Figure 2B; eFigure 13 in Supplement 1).

### *Shigella* Species

*Shigella* species isolates were mostly reported from travelers to sub-Saharan Africa (34%) and South America (24%), with the most common countries being Peru and India (eFigure 9 in Supplement 1). A single case of XDR *Shigella* species was reported from a traveler visiting the Caribbean region in 2022 and was treated with ertapenem as an outpatient.

**Figure 3.**
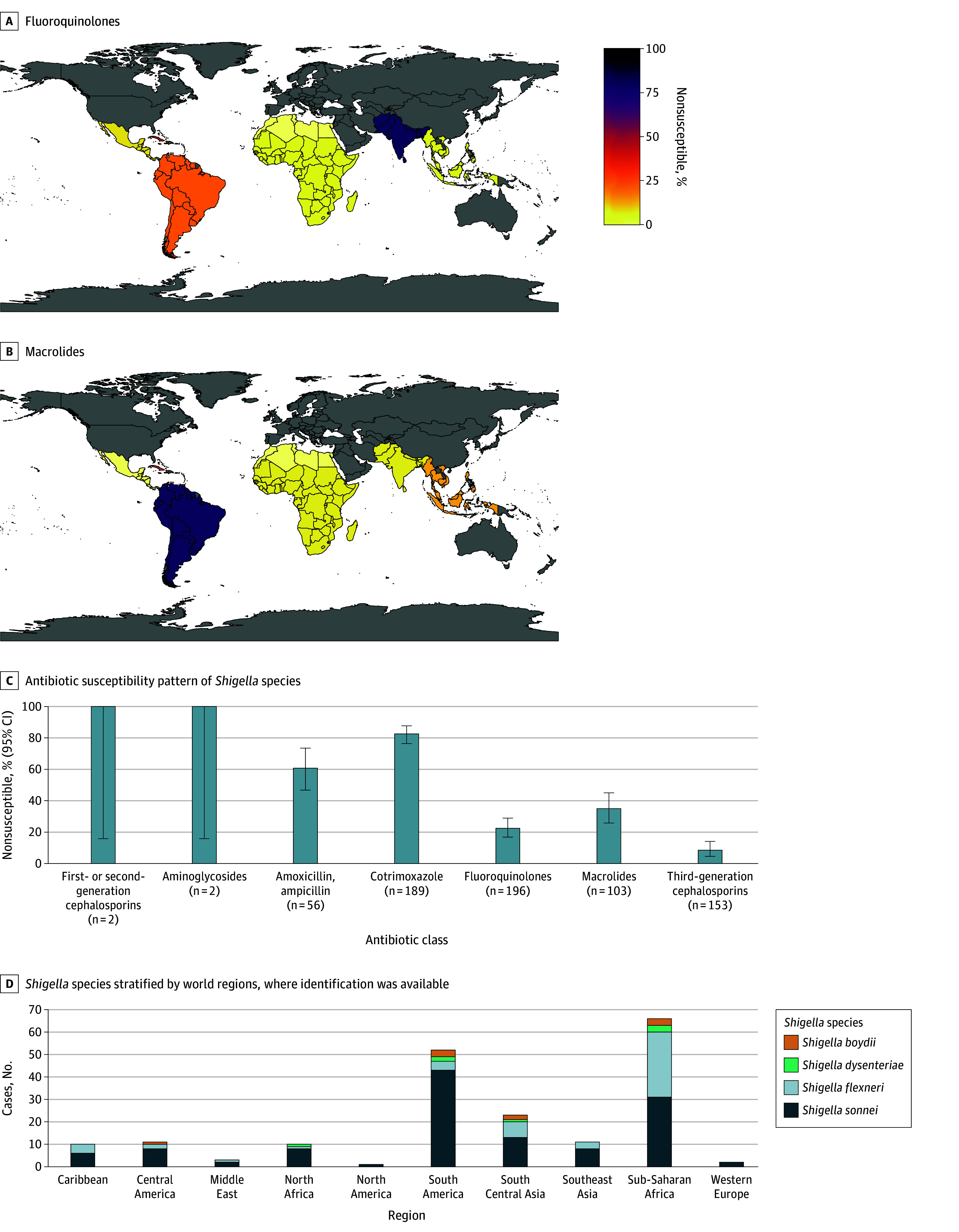
*Shigella* Species Nonsusceptibility to Antibiotics by World Region Maps show nonsusceptibility to fluoroquinolones (A) and macrolides (B). Only regions with 10 or more isolates with antimicrobial susceptibility test results are included. Middle East (n = 3), Western Europe (n = 3), and North America (n = 2) are not included. Panel C shows antibiotic susceptibility pattern of *Shigella* species to different antibiotics (n = 215). Panel D shows *Shigella* species stratified by world regions, where species identification was available (n = 189).

For *Shigella* species isolates overall, 44 of 196 (22%; 95% CI, 17%-29%) were fluoroquinolone nonsusceptible, 36 of 103 (35%; 95% CI, 26%-45%) were nonsusceptible to macrolides, and 13 of 153 (8%; 95% CI, 5%-14%) were nonsusceptible to third-generation cephalosporins (Figure 3; eTable 5 in Supplement 1). Notably, there was a high level of nonsusceptibility to fluoroquinolones (19 of 24 isolates [79%; 95% CI, 58%-93%]) in cases acquired in South Central Asia (Figure 3A; eFigure 14 in Supplement 1). Conversely, the highest levels of nonsusceptibility to macrolides were in cases acquired in South America (29 of 37 isolates [78%; 95% CI, 62%-90%) (Figure 3B; eFigure 15 in Supplement 1).

From a total of 215 *Shigella* cases, 189 (88%) had species identification available. There were 122 cases (65%) of *S sonnei*, 51 cases (27%) of *S flexneri*, 9 cases (5%) of *S boydii*, and 7 cases (4%) of *S dysenteriae. S sonnei* was the predominant species of *Shigella* in all regions. *S boydii* was isolated from cases acquired in sub-Saharan Africa (3 of 66 cases [5%]), South America (3 of 52 cases [6%]), Central America (1 of 11 cases [9%]), and South Central Asia (2 of 23 cases [9%]). *S flexneri* was isolated from travelers to sub-Saharan Africa (29 of 66 cases [44%]), South Central Asia (7 of 23 cases [30%]), South America (4 of 52 cases [8%]), the Caribbean (4 of 10 cases [40%]), Southeast Asia (3 of 11 cases [27%]), Central America (2 of 11 cases [18%]), Middle East (1 of 3 cases [33%]), and North Africa (1 of 10 cases [10%]). *S dysenteriae* was isolated from travelers to sub-Saharan Africa (3 of 66 cases [5%]) and South America (2 of 52 cases [4%]), with 1 additional case each being isolated from patient who had traveled to South Central Asia and North Africa (Figure 3D).

### Diarrheagenic *E coli*

**Figure 4.**
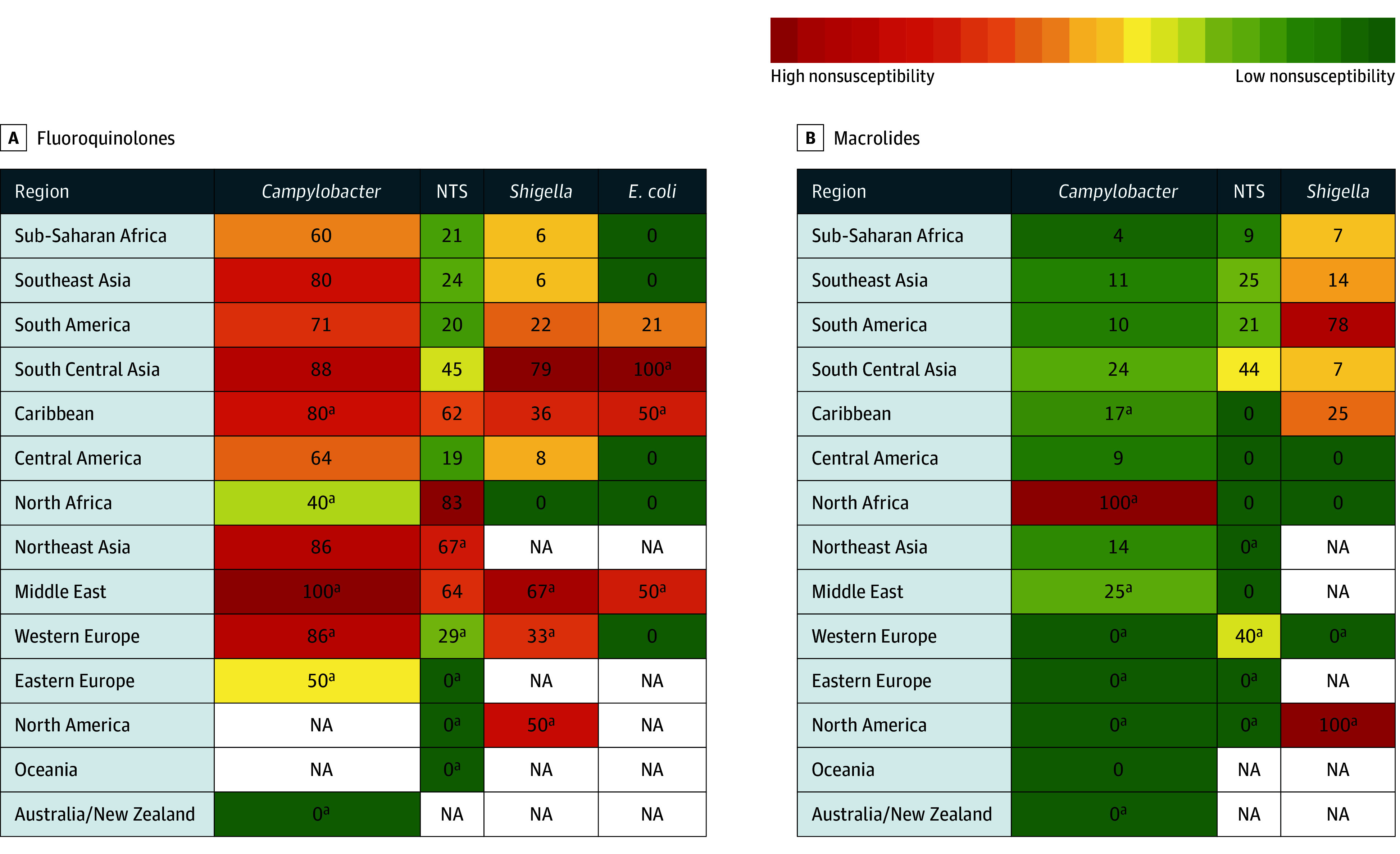
Heat Map of the Percentage Nonsusceptibility to Fluoroquinolones and Macrolides for Each of the Diarrheal Pathogens by World Regions NTS indicates nontyphoidal *Salmonella* species. ^a^Denotes regions with less than or equal to 10 isolates reported from the region.

The majority of diarrheagenic *E coli* cases with AST data available (43 of 75 cases [57%]) were reported from a single site (Lima, Peru) in South America. There were 58 cases (80%) of enteroaggregative, enteropathogenic, or enteroinvasive *E coli*, 9 cases (12%) Shiga toxin–producing *E coli* (enterohemorrhagic *E coli* or verocytotoxin-producing *E coli*, including *E coli* 0157:H7), and 6 cases (8%) of enterotoxigenic *E coli* (ETEC). Overall, fluoroquinolone nonsusceptibility was 12 of 66 isolates (18%; 95% CI, 10%-30%), and third-generation cephalosporin nonsusceptibility was 9 of 70 isolates (13%; 95% CI, 6%-23%) (eFigure 16 in Supplement 1). Fluoroquinolone nonsusceptibility in South America was noted in 9 of 43 isolates (21%; 95% CI, 10%-36%), with a single nonsusceptible isolate from South Central Asia. Susceptibility to macrolides was not determined (Figure 4; eTable 6 in Supplement 1).

Analysis by temporal trends, regions, and for visiting friends and relatives are shown in eFigures 17, 18, and 19 in Supplement 1. eTable 7 in Supplement 1 shows missing data, and eTable 8 in Supplement 1 shows analysis excluding the Lima site. See the eAppendix in Supplement 1 for supplemental results and discussion.

## Discussion

Leveraging a multicontinent, multisite tropical medicine surveillance network, in this cross-sectional study we examined the AST profile of enteropathogens isolated from 859 travelers to 103 destination countries. We found regional variations in demonstrated nonsusceptibility to fluoroquinolones and macrolides among the most common bacterial causes of acute TD.

With increasing use of culture-independent methods for the diagnosis of diarrheal illness, where antimicrobial sensitivity data are unavailable, tracking geographic antimicrobial resistance patterns will offer guidance to clinicians and policymakers regarding empiric therapy for treatment of moderate-to-severe TD.^9,14^ We demonstrate high rates of fluoroquinolone nonsusceptibility to diarrheal pathogens in several regions of the world.^6^ We also identified regions with high rates of macrolide nonsusceptibility, which is particularly concerning since macrolides are often the first-line agents used for empiric therapy provided to travelers. For example, for *Campylobacter *species, we found high rates of nonsusceptibility for fluoroquinolones in South Central Asia (88%) and Southeast Asia (80%), and lower but still concerning rates of nonsusceptibility for macrolides (24% and 11%, respectively).

*Shigella *species are a common cause of diarrhea among both travelers and residents of low-income and middle-income countries worldwide (Figure 3). We found a high proportion (78%) of macrolide nonsusceptibility in *Shigella* isolates among travelers to South America despite resistance first emerging in Southeast Asia.^18^ This prompts consideration of empiric use of third-generation cephalosporins in hospitalized patients (especially in those returning from South America) with *Shigella*, while susceptibility testing is pending. We show that across continents, most TD cases caused by *Shigella* species were caused by *S sonnei*.^19^ This is notable given the global rise of XDR *S sonnei*, so far mostly reported among men who have sex with men.^20,21^ The AST pattern observed in the diarrheagenic bacteria detailed here frequently differed from the antibiotics usually recommended for initial management of diarrhea in the travelers’ countries of origin.^22^ Notably, *Shigella* can be transmitted person-to-person, and antibiotic therapy can reduce disease transmission, which is important for some groups (eg, travelers who are young children, food handlers, and men who have sex with men).

This report elucidates the geographic variation of diarrheal disease according to cause and AST pattern, which might be useful for treatment guidelines. The top world regions represented in our study were sub-Saharan Africa and Southeast Asia, regions whose residents have the highest diarrhea burden globally.^23^ Our findings are consistent with other multisite studies of TD. In the Global Travelers’ Diarrhea study of US military and adult travelers, *E coli* was the most common cause. *Campylobacter* and *Salmonella* species rates were highest in Southeast, East, Southern, and Central Asia.^18,24^

With rising global antimicrobial resistance, AST surveillance across regions and populations is essential for guiding effective treatment. The use of rapid, culture-independent diagnostics without confirmatory testing risks delays antimicrobial resistance detection and appropriate empiric therapy. We recommend continued stool culture and susceptibility testing in international travelers to guide care and track global resistance trends for guideline updates.^25,26^ Emerging genomic tools may soon allow rapid susceptibility prediction.^27^ Judicious antimicrobial use in travelers is especially important, as antibiotic exposure during travel increases colonization with resistant organisms.^28^

Although there is limited evidence associating in vitro AST results with treatment outcomes for acute diarrhea,^29^ given the high nonsusceptibility rates for fluoroquinolones in *Campylobacter* and *Shigella* species, it is reasonable to continue to recommend macrolides for TD originating or occurring in South Central Asia. Similarly, in sub-Saharan Africa, where *Campylobacter* infections were a common cause of TD, macrolides are preferred given that 60% of isolates were nonsusceptible to fluoroquinolones. In South America, where there is 78% nonsusceptibility to macrolides in *Shigella* species, as well as 71% nonsusceptibility to fluoroquinolones in *Campylobacter* species, patients receiving empiric therapy may need closer monitoring for persistent or worsening symptoms and possible broader antibiotic coverage.

Macrolides are recommended for all geographic regions for all 4 organisms apart from *Shigella* species in South America, where fluoroquinolones are more effective. Culture is truly necessary in the centers where this is feasible, and culture media are available. Cost-effectiveness should also be considered especially in during-travel sites located in low-income and middle-income countries.

### Limitations

Although our findings provide new insights into antimicrobial nonsusceptibility in travelers, several limitations exist. GeoSentinel data come from specialized clinics and a convenience sample, not representing all travelers. The network includes only numerator data, and most sites are in high-income countries, likely missing cases from low-income regions. Data collection at GeoSentinel sites is discretionary, not systematic, potentially resulting in underreporting and bias. Included patients may reflect more severe illness, as self-treated TD cases were not captured. This may affect generalizability as AST may only have been done for patients with moderate-to-severe diarrhea who present to specialized centers that regularly perform cultures. AST reporting varies by site and is based on phenotypic rather than genotypic methods. Pooling resistant and intermediate results into 1 nonsusceptible category may have overestimated resistance. Testing followed local guidelines, limiting consistency. Although most laboratories routinely culture stool samples for *Salmonella*, *Shigella*, and *Campylobacter* species, culturing for *E coli* is not routine, and the majority of our *E coli* cases were reported from a single site in Peru. This may implicate that one cannot generalize the *E coli* data to portray data across the world. Another limitation is the lack of data on prior antibiotic use. However, short self-treatment courses are unlikely to drive resistance, even with prior use. Only 6 cases of ETEC were present in this study, perhaps owing to fewer cultures performed for *E coli*, while ETEC is one of the most common causes of TD. Not all sites perform AST for all antibiotics of interest for the diarrheagenic pathogens, hence leading to missing data, which may also affect the results.

## Conclusions

In this cross-sectional study of AST patterns in TD from GeoSentinel, our report leverages a large clinical network to report the AST profile of bacterial pathogens causing acute diarrhea in travelers to over 100 destination countries. Our findings have the potential to provide critical information for both the selection of standby empiric antibiotics for self-management of TD, and the empiric treatment of acute diarrhea in travelers, by travel medicine and primary care health care practitioners. Our study findings also demonstrate the importance of surveillance of culture and susceptibility testing in TD in order to identify geographic patterns of resistance.

## Notes

Supplement 1. eFigure 1. Flowchart showing extraction profile for *Campylobacter* species, non-typhoidal *Salmonella* species, *Shigella* species and *Escherichia coli* included in the GeoSentinel 2015-2022 analysis eTable 1. AST methods according to the sites with available data (n=25) eTable 2. Comparison of tested vs. untested isolates eFigure 2. GeoSentinel sites that reported diarrheal pathogens with available antibiotic susceptibility data, with each dot representing the GeoSentinel site and the color intensity representing the number of cases reported from the site eFigure 3. GeoSentinel sites that contributed to the AST data for diarrheal pathogens eFigure 4. Countries of exposure of all diarrheal pathogens with available antibiotic susceptibility data eFigure 5. Diarrheagenic organisms in all cases and those with antimicrobial susceptibility data available eFigure 6. Top ten countries of exposure of diarrheal pathogens having AST data available eFigure 7. Primary reason for travel in all patients with diarrheal pathogens having AMR data eFigure 8. Type of care at hospital based on diarrheal pathogens eFigure 9. Top three countries of exposure for each of the diarrheal pathogens eTable 3. Antimicrobial susceptibility data for *Campylobacter* species eTable 4. Antimicrobial susceptibility data for non-typhoidal *Salmonella* species eTable 5. Antimicrobial susceptibility data for *Shigella* species eTable 6. Antimicrobial susceptibility data for diarrheagenic *E. coli* species eFigure 10. Non-susceptibility pattern of *Campylobacter* species to fluoroquinolones stratified by world regions eFigure 11. Non-susceptibility pattern of *Campylobacter* species to macrolides stratified by world regions eFigure 12. Non-susceptibility pattern of non-typhoidal *Salmonella* species to fluoroquinolones stratified by world regions eFigure 13. Non-susceptibility pattern of non-typhoidal *Salmonella* species to macrolides stratified by world regions eFigure 14. Antibiotic susceptibility pattern of *Shigella* species to fluoroquinolones stratified by world regions eFigure 15. Antibiotic susceptibility pattern of *Shigella* species to macrolides stratified by world regions eFigure 16. Antibiotic susceptibility pattern of diarrheagenic *E. coli* to different antibiotics (n=75) eFigure 17. Temporal trends of AST of fluoroquinolones from 2015-2022 by bacteria eFigure 18. Temporal trends of AST of macrolides from 2015-2022 by bacteria eFigure 19. Temporal trends of AST of third generation cephalosporins from 2015-2022 by bacteria eTable 7. Table showing missing data patterns i.e. unknown/not done/not reported by organism, antibiotic class and region eTable 8. Diarrheagenic *E. coli* non-susceptibility excluding the Lima site eAppendix. Supplemental results and discussion

Supplement 2. Nonauthor Collaborators. GeoSentinel Surveillance Network Collaborators

Supplement 3. Data Sharing Statement
