# Errors in Byline, Abstract, and Methods

**DOI:** 10.1001/jamanetworkopen.2025.59709

**Published:** 2026-01-27

In the Original Investigation titled “GeoSentinel Analysis of Travelers’ Diarrhea Antimicrobial Resistance Patterns,”^1^ published December 22, 2025, there was an error in the byline; Daniel T. Leung’s middle initial was missing. There was also an error in the abstract. The word *Shigella* was missing in 2 places in the final sentence, which should read, “Of note, 19 of 24 *Shigella* isolates (79%; 95% CI, 58%-93%) were nonsusceptible to fluoroquinolones among travelers to South Central Asia, and 29 of 37 *Shigella* isolates (78%; 95% CI, 62%-90%) were nonsusceptible to macrolides among travelers to South America.” Additionally, a sentence in the Methods was missing the word “recommended” in 2 places and should read, “Most sites used either Clinical and Laboratory Standards Institute–recommended or European Committee on Antimicrobial Susceptibility Testing–recommended American Type Culture Collection strains of bacteria for external and internal quality control and had periodic quality control testing.” This article has been corrected.^1^
