# Supplementary material for: GeoSentinel Analysis of Travelers’ Diarrhea Antimicrobial Resistance Patterns
Source: JAMA Netw Open. 2025 Dec 22;8(12):e2551089. doi: 10.1001/jamanetworkopen.2025.51089 (PMC12723550; doi:10.1001/jamanetworkopen.2025.51089)
Supplement: Supplement 1. — eFigure 1. Flowchart showing extraction profile for Campylobacter species, non-typhoidal Salmonella species, Shigella species and Escherichia coli included in the GeoSentinel 2015-2022 analysis eTable 1. AST methods according to the sites with available data (n=25) eTable 2. Comparison of tested vs. untested isolates eFigure 2. GeoSentinel sites that reported diarrheal pathogens with available antibiotic susceptibility data, with each dot representing the GeoSentinel site and the color intensity representing the number of cases reported from the site eFigure 3. GeoSentinel sites that contributed to the AST data for diarrheal pathogens eFigure 4. Countries of exposure of all diarrheal pathogens with available antibiotic susceptibility data eFigure 5. Diarrheagenic organisms in all cases and those with antimicrobial susceptibility data available eFigure 6. Top ten countries of exposure of diarrheal pathogens having AST data available eFigure 7. Primary reason for travel in all patients with diarrheal pathogens having AMR data eFigure 8. Type of care at hospital based on diarrheal pathogens eFigure 9. Top three countries of exposure for each of the diarrheal pathogens eTable 3. Antimicrobial susceptibility data for Campylobacter species eTable 4. Antimicrobial susceptibility data for non-typhoidal Salmonella species eTable 5. Antimicrobial susceptibility data for Shigella species eTable 6. Antimicrobial susceptibility data for diarrheagenic E. coli species eFigure 10. Non-susceptibility pattern of Campylobacter species to fluoroquinolones stratified by world regions eFigure 11. Non-susceptibility pattern of Campylobacter species to macrolides stratified by world regions eFigure 12. Non-susceptibility pattern of non-typhoidal Salmonella species to fluoroquinolones stratified by world regions eFigure 13. Non-susceptibility pattern of non-typhoidal Salmonella species to macrolides stratified by world regions eFigure 14. Antibiotic susceptibility pattern of Shigella specie [file jamanetwopen-e2551089-s001.pdf]

Supplemental Online Content

Amatya B, Pandey P, McGuinness SL, et al; GeoSentinel Surveillance Network. GeoSentinel analysis of travelers' diarrhea antimicrobial resistance patterns. *JAMA Netw Open*. 2025;8(12):e2551089. doi:10.1001/jamanetworkopen.2025.51089

eFigure 1. Flowchart showing extraction profile for *Campylobacter* species, non-typhoidal *Salmonella* species, *Shigella* species and *Escherichia coli* included in the GeoSentinel 2015-2022 analysis
eTable 1. AST methods according to the sites with available data (n=25)
eTable 2. Comparison of tested vs. untested isolates
eFigure 2. GeoSentinel sites that reported diarrheal pathogens with available antibiotic susceptibility data, with each dot representing the GeoSentinel site and the color intensity representing the number of cases reported from the site
eFigure 3. GeoSentinel sites that contributed to the AST data for diarrheal pathogens
eFigure 4. Countries of exposure of all diarrheal pathogens with available antibiotic susceptibility data
eFigure 5. Diarrheagenic organisms in all cases and those with antimicrobial susceptibility data available
eFigure 6. Top ten countries of exposure of diarrheal pathogens having AST data available
eFigure 7. Primary reason for travel in all patients with diarrheal pathogens having AMR data
eFigure 8. Type of care at hospital based on diarrheal pathogens
eFigure 9. Top three countries of exposure for each of the diarrheal pathogens
eTable 3. Antimicrobial susceptibility data for *Campylobacter* species
eTable 4. Antimicrobial susceptibility data for non-typhoidal *Salmonella* species
eTable 5. Antimicrobial susceptibility data for *Shigella* species
eTable 6. Antimicrobial susceptibility data for diarrheagenic *E. coli* species
eFigure 10. Non-susceptibility pattern of *Campylobacter* species to fluoroquinolones stratified by world regions
eFigure 11. Non-susceptibility pattern of *Campylobacter* species to macrolides stratified by world regions
eFigure 12. Non-susceptibility pattern of non-typhoidal *Salmonella* species to fluoroquinolones stratified by world regions
eFigure 13. Non-susceptibility pattern of non-typhoidal *Salmonella* species to macrolides stratified by world regions
eFigure 14. Antibiotic susceptibility pattern of *Shigella* species to fluoroquinolones stratified by world regions
eFigure 15. Antibiotic susceptibility pattern of *Shigella* species to macrolides stratified by world regions
eFigure 16. Antibiotic susceptibility pattern of diarrheagenic *E. coli* to different antibiotics (n=75)
eFigure 17. Temporal trends of AST of fluoroquinolones from 2015-2022 by bacteria
eFigure 18. Temporal trends of AST of macrolides from 2015-2022 by bacteria

© 2025 Amatya B et al. *JAMA Network Open*.

eFigure 19. Temporal trends of AST of third generation cephalosporins from 2015-2022 by bacteria
eTable 7. Table showing missing data patterns i.e. unknown/not done/not reported by organism, antibiotic class and region
eTable 8. Diarrheagenic *E. coli* non-susceptibility excluding the Lima site
eAppendix. Supplemental results and discussion

This supplemental material has been provided by the authors to give readers additional information about their work.

© 2025 Amatya B et al. *JAMA Network Open*.

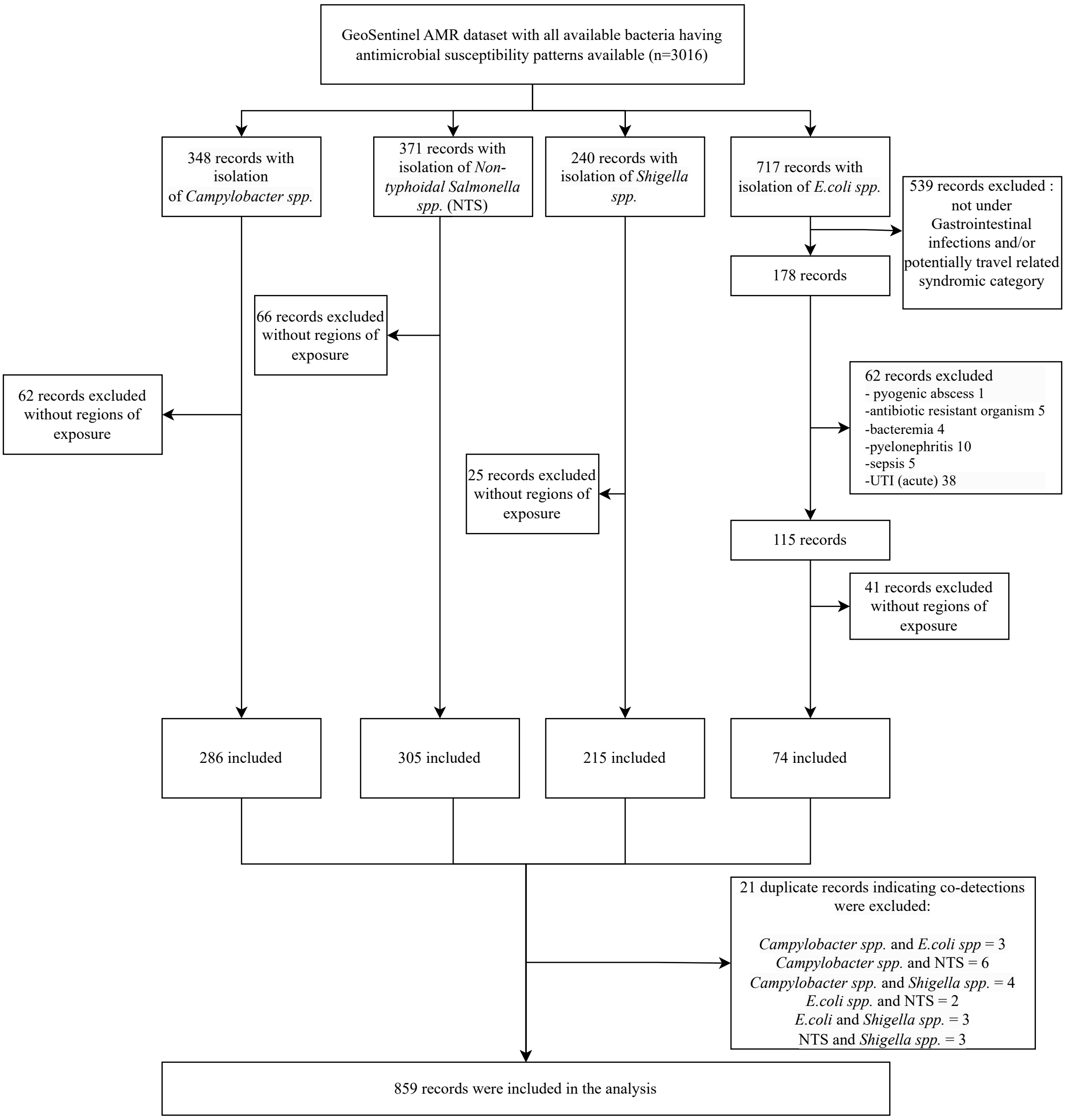

eFigure 1. Flowchart showing extraction profile for *Campylobacter* species, non-typhoidal *Salmonella* species, *Shigella* species and *Escherichia coli* included in the GeoSentinel 2015-2022 analysis

© 2025 Amatya B et al. *JAMA Network Open*.

eTable 1. AST methods according to the sites with available data (n=25).

Site	AST method	Breakpoint	Please describe the quality control procedures for AST performed at your site.
Aarhus	Disk diffusion	EUCAST	Using EUCAST ATCC strains
Antwerp	Disk diffusion; MIC (Minimum Inhibitory Concentration)	EUCAST	**Internal quality control**<br>Every four weeks, an antibiogram is performed on the following three reference strains:<br>E. coli ATCC 25922, S. aureus ATCC 29213, and S. pneumoniae ATCC 49619.<br>Every four months, the following strains are tested for susceptibility:<br>P. aeruginosa ATCC 27853, E. faecalis ATCC 29212, S. aureus NCTC 12493 MRSA, S. aureus ATCC 29213, E. coli ATCC 35218, K. pneumoniae ATCC 700603, and E. coli ATCC 25922.<br>The antibiotics being tested and the criteria the results must meet are listed on the form Internal Quality Control for Susceptibility Testing in Bacteriology (EUCAST criteria). If a result does not meet the specified criteria, the test is repeated, the clinical biologist is informed, and the relevant antibiotic is no longer used for determining susceptibility of organisms until the clinical biologist gives approval.<br>First-line controls are rotated among all MLTs (Medical Laboratory Technologists).<br><br>**Lot controls**<br>An incoming inspection (lot control) is also performed on all delivered antibiotic discs and E-tests. A new lot is only used after it has been tested and approved. Results are recorded on the form Internal Quality Control for Susceptibility Testing in Bacteriology.<br><br>**Between reader variability controls**<br>Every 3 months, a tuning is performed for the 3 reference strains (E. coli ATCC 25922, S. aureus ATCC 29213, and S. pneumoniae ATCC 49619), to be read by the 4 MLTs in bacteriology.<br>Criteria: maximum 3 mm difference or 2 dilutions for E-tests. Results are recorded on Tuning of the 3 reference strains.<br><br>**External quality control**<br>External quality scheme Sciensano 3x/ year and UK Neqas 1x/year. These are not always bacteria related to travelers' diarrhea.

© 2025 Amatya B et al. *JAMA Network Open*.

Bucharest	Automated systems; Disk diffusion; MIC (Minimum Inhibitory Concentration); MICRODILUTIONS IN BROTH- MICRONAUT	EUCAST	Internal and external quality control with ATCC strains, four times per year (for the external control) and weekly (for internal control).<br>We follow the RENAR procedures (quality control systems for laboratories).
Bangkok	Disk diffusion; Next year, we plan to move to MIC and automated system	CLSI	We use External Quality Assurance system from Department of Medical Science, Ministry of Public Health. We need to certify every 4 months (three times a year)
Bordeuax	Automated systems; Disk diffusion; MIC (Minimum Inhibitory Concentration)	CA-SFM	Periodic EQA (at least yearly, depending on the pathogen) + more regular internal quality controls, according to the COFRAC accreditation framework and procedures
Calgary	Automated systems; Disk diffusion; MIC (Minimum Inhibitory Concentration)	CLSI	Molecular panel for TD screening, with culture confirmation then microbroth.
Chile	Automated systems; Disk diffusion; MIC (Minimum Inhibitory Concentration)	CLSI	There is a comprehensive 29-page document of guidelines for quality control of antimicrobial susceptibility procedures. It is called Quality control for antimicrobial susceptibility. In summary, quality controls are performed at least once a week, particularly for techniques that are used routinely. The lab is also subject to external quality control such as the College of American Pathologists-CAP and with the ISP-PEEC (National Public Health Institute)
Geneva	Automated systems; Disk diffusion; MIC (Minimum Inhibitory Concentration)	Both	Internal quality control once a week for disk diffusion & every month for antibiogram by E-test and serial dilution. With EUCAST strains
Lima	Automated systems; Disk diffusion; MIC (Minimum Inhibitory Concentration)	CLSI	None
Liverpool School of Tropical Medicine	Disk diffusion; MIC (Minimum Inhibitory Concentration)	EUCAST	Weekly QC check. EUCAST recommended QC procedures followed. Includes control strains used for Enterobacterales (incl E coli ATCC 35218 and ATCC 25922) and Campylobacter (ATCC 33560 C. jejuni), tested using the same methodology/range of discs used for clinical isolates, with results documented in a spreadsheet maintained on a secure server. The laboratory is externally accredited by UKAS (United Kingdom Accreditation Service)
Madrid	Automated systems; MIC (Minimum Inhibitory Concentration)	EUCAST	At our site AST follows EUCAST guidelines under an ISO 15189-accredited quality system. QC strains (e.g., E. coli ATCC 25922, S. aureus ATCC 29213) are tested regularly and with each new batch of media or antibiotic disks. Results are reviewed daily, deviations investigated, and corrective actions documented. The laboratory also participates in SEIMC and INSTAND external quality assessment programs to ensure accuracy and reliability.

© 2025 Amatya B et al. *JAMA Network Open*.

Melbourne	Automated systems; Disk diffusion; MIC (Minimum Inhibitory Concentration)	CLSI	Quality Control is performed as per CLSI guidelines.
Montreal	Automated systems; Disk diffusion; MIC (Minimum Inhibitory Concentration)	CLSI	We use ATCC strains as given in CLSI M100-Ed35. Quality controls are done daily and documented. All data from QC worksheets are entered on a daily basis into an Antibiotic QC trend electronic spreadsheet document. This spreadsheet is reviewed by the quality ACT on a weekly basis.
Negrar	Automated systems; Disk diffusion; MIC (Minimum Inhibitory Concentration)	EUCAST	Our laboratory performs rigorous quality control for all AST, including daily checks with reference strains (e.g., E. coli ATCC 25922, S. aureus ATCC 29213), verification of media and reagents, and regular instrument calibration. Results are monitored internally and through external proficiency testing to ensure accuracy and reliability.
New York West	Automated systems; Disk diffusion; MIC (Minimum Inhibitory Concentration)	CLSI	MicroScan AST: weekly controls<br><br>e-tests and Kirby-Bauer discs: weekly or day of use
Stockholm	Automated systems; Disk diffusion; MIC (Minimum Inhibitory Concentration)	EUCAST	The clinical microbiological laboratory participates in a European (international?) collaboration/system by regularly receiving control samples. I don´t have the details on the frequency and controls used today. If needed, I could ask those responsible at the laboratory - but it may take some time to receive the answers.
Brescia	Automated systems; Disk diffusion; MIC (Minimum Inhibitory Concentration)	EUCAST	All antibiotic panels used in semi-automated systems are tested with ATCC strains (bacterial strains with a known phenotypic profile).
Tokyo	Automated systems; e-test; MIC (Minimum Inhibitory Concentration)	CLSI	Using standard strains from Microbiologics, we measure antimicrobial susceptibility at least once a week to confirm that results remain within the reference range. This is also performed whenever the batch of test reagents changes.
Tan Tock Seng Hospital	Automated systems; Disk diffusion; MIC (Minimum Inhibitory Concentration)	CLSI	Quality Control as required by CLSI.
Utah, Salt Lake City	Automated systems; Disk diffusion; MIC (Minimum Inhibitory Concentration)	CLSI	Quite complicated and changed over the years
Winnipeg	Automated systems; Disk diffusion; MIC (Minimum Inhibitory Concentration)	CLSI	Unknown
Yokohama	Automated systems	CLSI	Unknown

© 2025 Amatya B et al. *JAMA Network Open*.

Nairobi	Automated systems	Both	Weekly review by lab, monthly review by AMS
	Automated systems; Disk diffusion; MIC (Minimum Inhibitory Concentration)	CLSI	Standard per US clinical labs. Since the merger in the Beth Israel Lahey Health Network, our Micro Lab is based in another network hospital.
	Automated systems; Disk diffusion; MIC (Minimum Inhibitory Concentration); Molecular testing	Both	ATCC strains according to CLSI recommendations. External quality control.

© 2025 Amatya B et al. *JAMA Network Open*.

eTable 2. Comparison of tested vs. untested isolates.

organism	Antibiotic class	Tested	Untested
Campylobacter	1st or 2nd Generation cephalosporins	7	NA
	Amoxicillin, Ampicillin	8	NA
	Chloramphenicol	1	NA
	betalactam/betalactamase inhibitor combination	1	NA
	cotrimoxazole	6	NA
	fluoroquinolones	274	8
	macrolides	255	27
Salmonella Species	1st or 2nd Generation cephalosporins	7	NA
	3rd generation cephalosporins	273	63
	Amoxicillin, Ampicillin	133	44
	Penicillin	1	NA
	Tigecycline	1	NA
	aminoglycosides	9	NA
	betalactam/betalactamase inhibitor combination	2	NA
	carbapenems	137	198
	cotrimoxazole	275	59
	fluoroquinolones	302	31
	macrolides	111	222
Shigella Species	1st or 2nd Generation cephalosporins	2	NA
	3rd generation cephalosporins	153	53
	Amoxicillin, Ampicillin	56	9
	Tigecycline	1	NA
	aminoglycosides	2	NA
	cotrimoxazole	189	18
	fluoroquinolones	196	11
	macrolides	103	103

© 2025 Amatya B et al. *JAMA Network Open*.

E. Coli	Chloramphenicol	NA	2
	Nitrofurantoin	NA	2
	macrolides	NA	2
	1st or 2nd Generation cephalosporins	2	NA
	3rd generation cephalosporins	70	NA
	4th generation cephalosporins	56	14
	Amoxicillin, Ampicillin	66	4
	Penicillin	2	NA
	Tigecycline	2	NA
	aminoglycosides	2	NA
	betalactam/betalactamase inhibitor combination	2	NA
	carbapenems	63	7
	cotrimoxazole	63	7
	fluoroquinolones	66	4
	polymyxins	38	32

© 2025 Amatya B et al. *JAMA Network Open*.

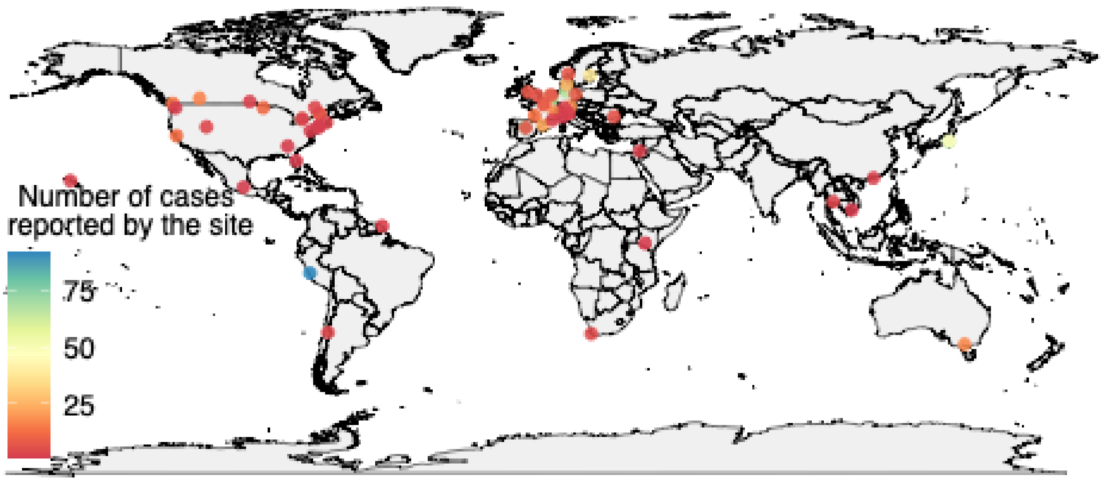

eFigure 2. GeoSentinel sites that reported diarrheal pathogens with available antibiotic susceptibility data, with each dot representing the GeoSentinel site and the color intensity representing the number of cases reported from the site.

****The Cambridge, UK site is no longer a part of the GeoSentinel Network but had contributed six cases to this analysis.**

*****The Cuernavaca, Mexico site is no longer a part of the GeoSentinel Network but had contributed one case to this analysis.**

******Ho Chi Minh City, Vietnam site is no longer a part of the GeoSentinel Network but had contributed one case to this analysis.**

******The Seattle, USA site also is no longer a part of the GeoSentinel Network but had contributed one case to this analysis.**

*******The Toronto, Canada site is no longer a part of the GeoSentinel Network but had contributed 16 cases to this analysis.**

********The Amsterdam, Netherlands site is no longer a part of the GeoSentinel Network but had contributed six cases to this analysis.**

© 2025 Amatya B et al. *JAMA Network Open*.

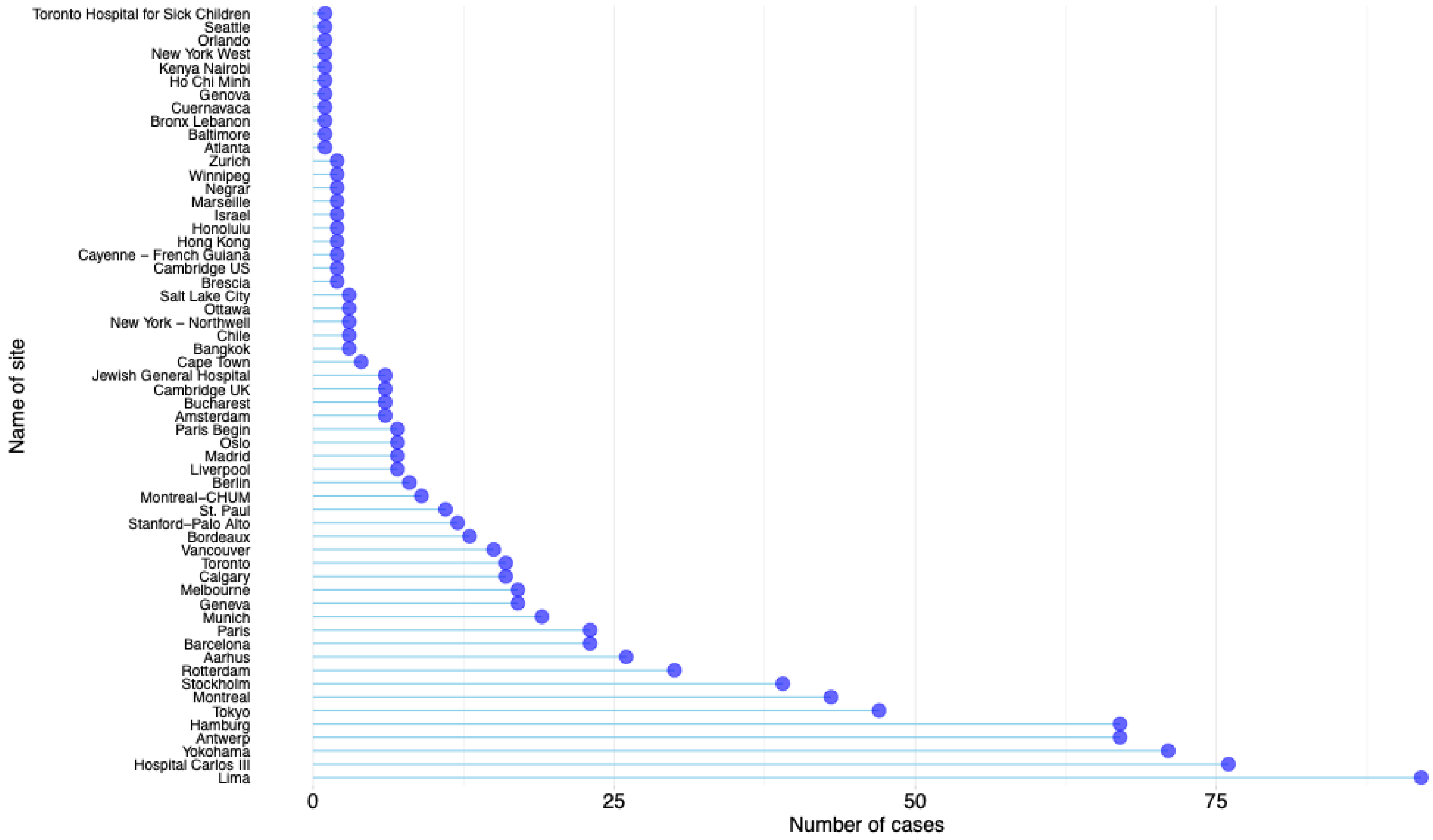

eFigure 3. GeoSentinel sites that contributed to the AST data for diarrheal pathogens.

© 2025 Amatya B et al. *JAMA Network Open*.

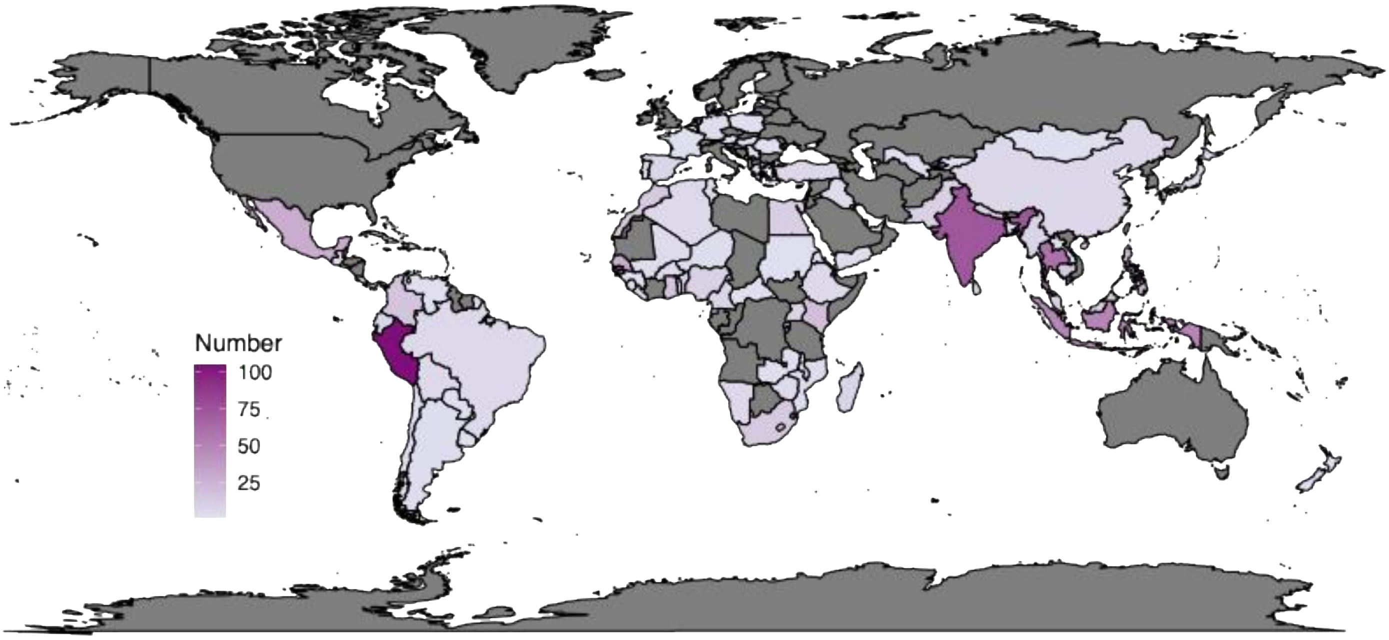

eFigure 4. Countries of exposure of all diarrheal pathogens with available antibiotic susceptibility data.

© 2025 Amatya B et al. *JAMA Network Open*.

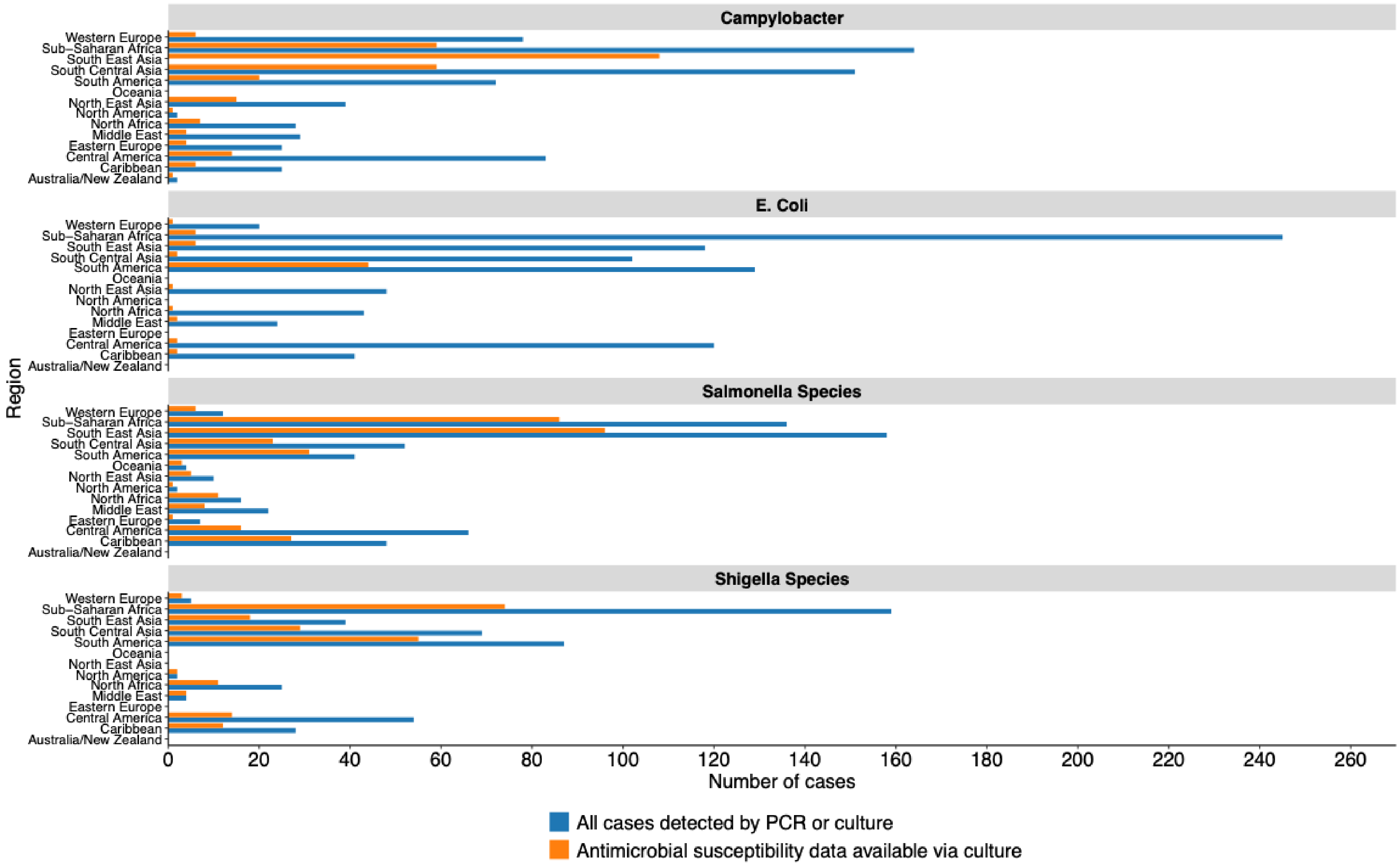

eFigure 5. Diarrheagenic organisms in all cases and those with antimicrobial susceptibility data available.

© 2025 Amatya B et al. *JAMA Network Open*.

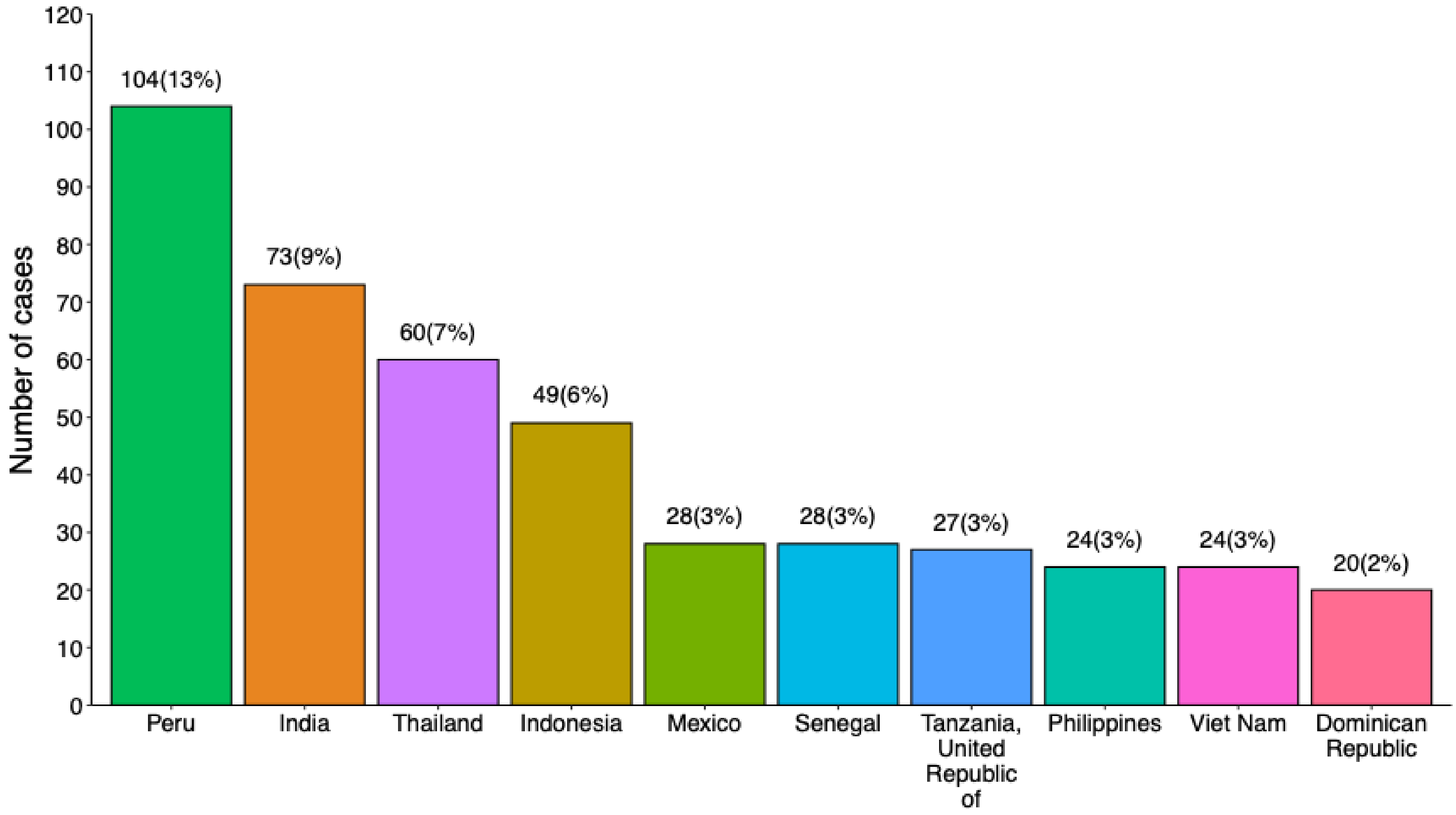

eFigure 6. Top ten countries of exposure of diarrheal pathogens having AST data available.

© 2025 Amatya B et al. *JAMA Network Open*.

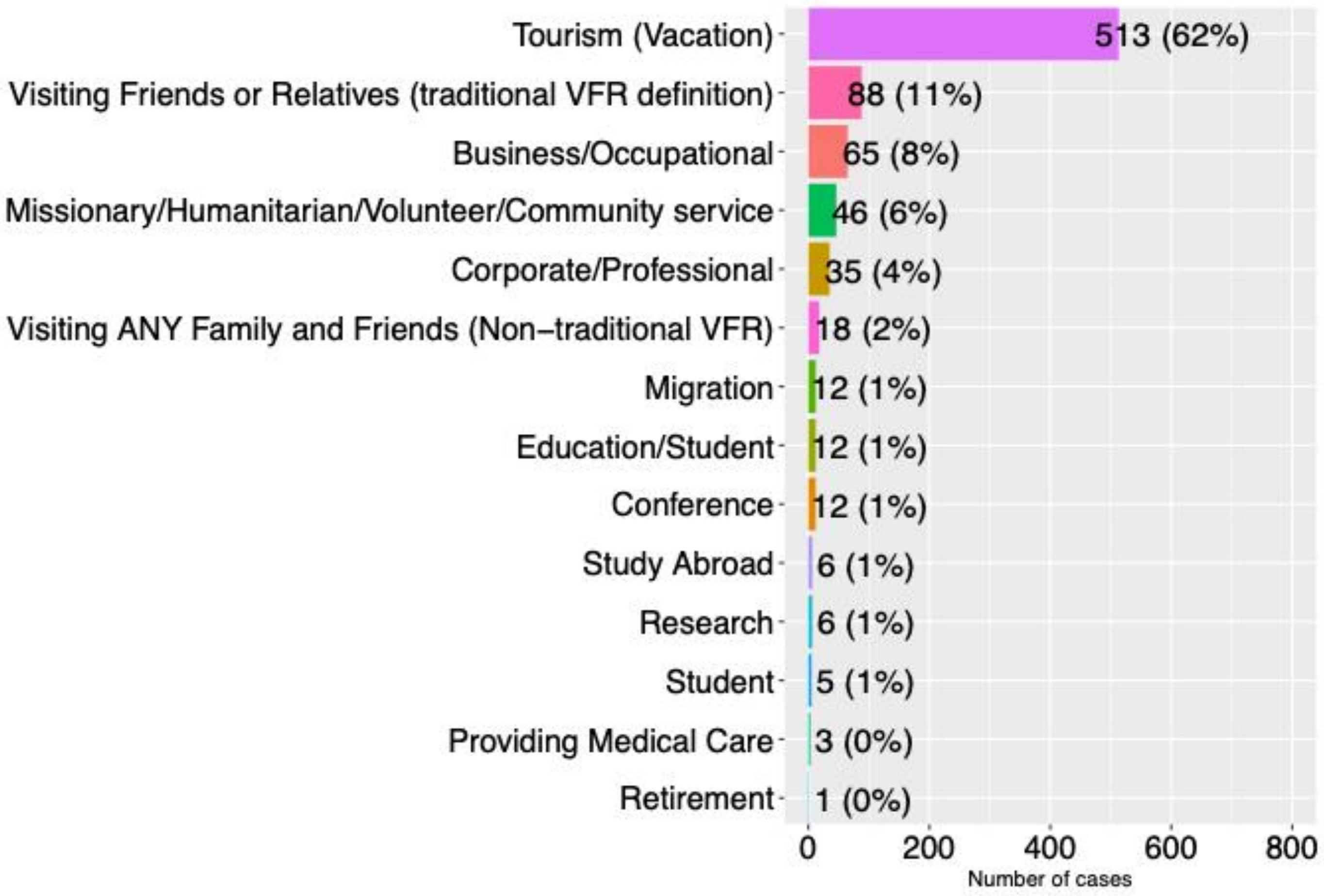

eFigure 7. Primary reason for travel in all patients with diarrheal pathogens having AMR data.
(Percentages may not round to 100%, due to rounding). There were 31 cases with missing travel reason and six were listed as "others".

© 2025 Amatya B et al. *JAMA Network Open*.

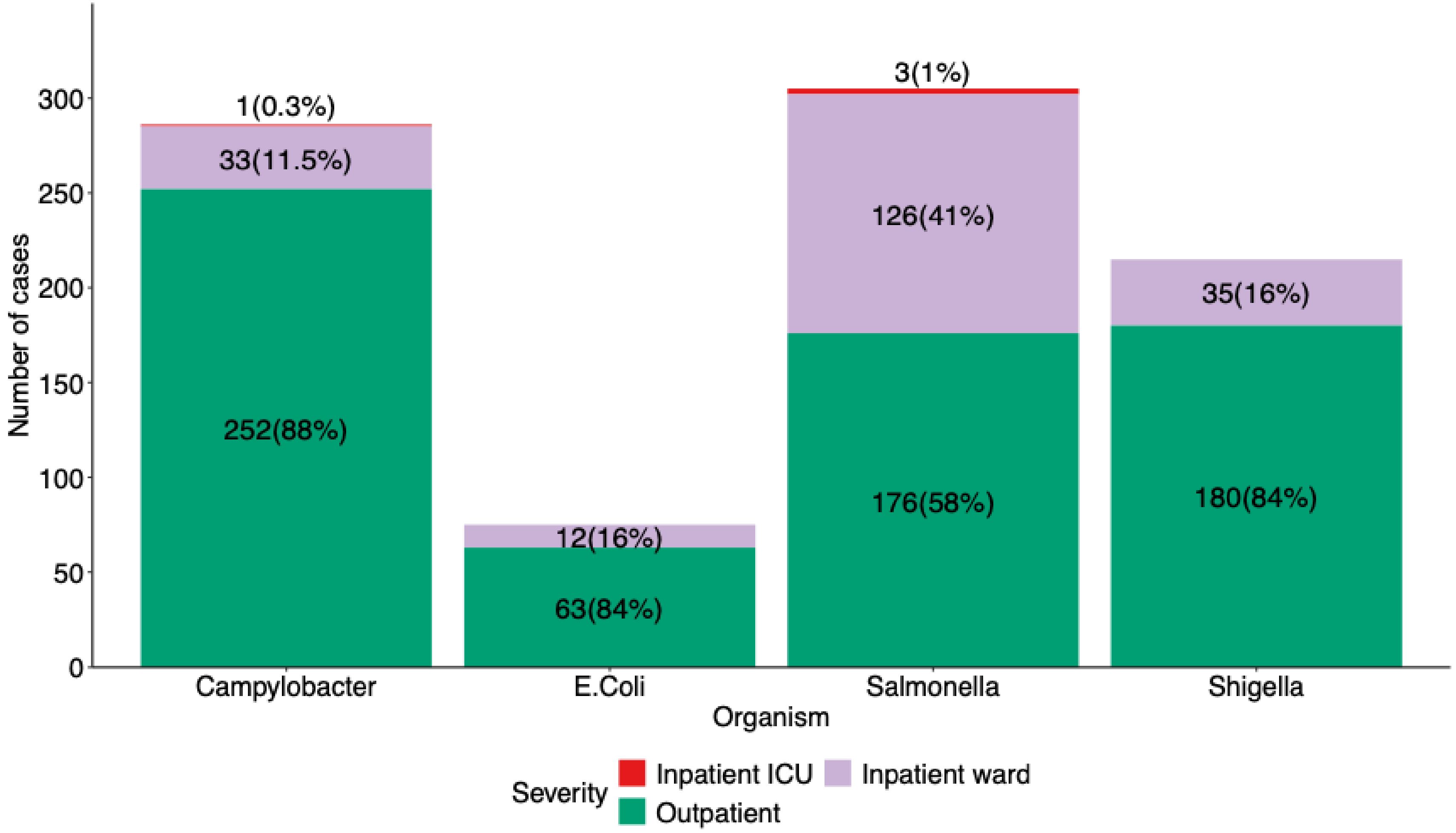

eFigure 8. Type of care at hospital based on diarrheal pathogens.

© 2025 Amatya B et al. *JAMA Network Open*.

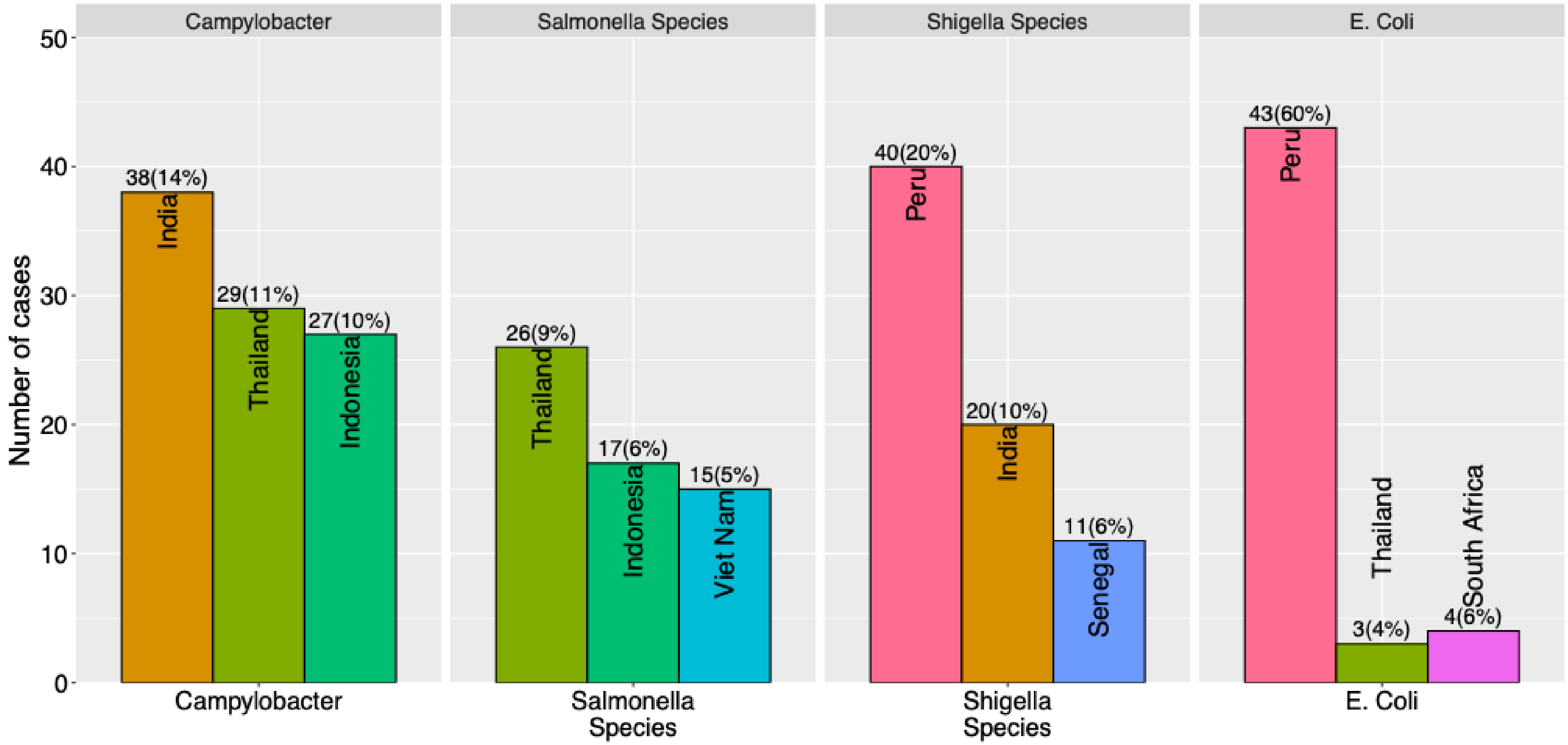

eFigure 9. Top three countries of exposure for each of the diarrheal pathogens.

© 2025 Amatya B et al. *JAMA Network Open*.

eTable 3. Antimicrobial susceptibility data for *Campylobacter* species.

Antibiotic class	Non-susceptible	Susceptible	Unknown/Not Done/Not Reported
Fluoroquinolone (e.g. Ciprofloxacin, Norfloxacin, Ofloxacin, Levofloxacin)	206	68	8
Macrolide (e.g. Azithromycin, Erythromycin, Clarithromycin)	30	225	27
Amoxicillin, Ampicillin	8	0	0
1st or 2nd Generation Cephalosporin: Cefazolin, Cephalexin, Cefadroxil, Cefoxitin, Cefuroxime, Cefotetan, or other	7	0	0
Cotrimoxazole (trimethoprim-sulfamethoxazole)	6	0	0
Betalactam/Betalactamase inhibitor combination: eg Ampicillin-Clavunate, Piperacillin-Tazobactam, or other	1	0	0
Chloramphenicol	1	0	0

© 2025 Amatya B et al. *JAMA Network Open*.

eTable 4. Antimicrobial susceptibility data for non-typhoidal *Salmonella* species.

Antibiotic class	Non-susceptible	Susceptible	Unknown/Not Done/Not Reported
1st or 2nd Generation Cephalosporin: Cefazolin, Cephalexin, Cefadroxil, Cefoxitin, Cefuroxime, Cefotetan, or other	7	0	0
3rd Generation Cephalosporin (e.g. Cefixime, Cefotaxime, Ceftriaxone, Ceftazidime)	15	258	63
Aminoglycosides: eg Gentamicin, Tobramycin, Amikacin	9	0	0
Amoxicillin, Ampicillin	37	96	44
Betalactam/Betalactamase inhibitor combination: eg Ampicillin-Clavunate, Piperacillin-Tazobactam, or other	2	0	0
Carbapenem (e.g. Imipenem, Meropenem, Ertapenem)	1	136	198
Cotrimoxazole (trimethoprim-sulfamethoxazole)	44	231	59
Fluoroquinolone (e.g. Ciprofloxacin, Norfloxacin, Ofloxacin, Levofloxacin)	96	206	31
Macrolide (e.g. Azithromycin, Erythromycin, Clarithromycin)	18	93	222
Penicillin	1	0	0
Tigecycline	1	0	0

© 2025 Amatya B et al. *JAMA Network Open*.

eTable 5. Antimicrobial susceptibility data for *Shigella* species.

Antibiotic class	Non-susceptible	Susceptible	Unknown/Not Done/Not Reported
1st or 2nd Generation Cephalosporin: Cefazolin, Cephalexin, Cefadroxil, Cefoxitin, Cefuroxime, Cefotetan, or other	2	0	0
3rd Generation Cephalosporin (e.g. Cefixime, Cefotaxime, Ceftriaxone, Ceftazidime)	13	140	53
Aminoglycosides: eg Gentamicin, Tobramycin, Amikacin	2	0	0
Amoxicillin, Ampicillin	34	22	9
Cotrimoxazole (trimethoprim-sulfamethoxazole)	156	33	18
Fluoroquinolone (e.g. Ciprofloxacin, Norfloxacin, Ofloxacin, Levofloxacin)	44	152	11
Macrolide (e.g. Azithromycin, Erythromycin, Clarithromycin)	36	67	103
Tigecycline	1	0	0

© 2025 Amatya B et al. *JAMA Network Open*.

eTable 6. Antimicrobial susceptibility data for diarrheagenic *E. coli* species.

Antibiotic class	Intermediate/Resistant	Sensitive	Unknown/Not Done/Not Reported
1st or 2nd Generation Cephalosporin: Cefazolin, Cephalexin, Cefadroxil, Cefoxitin, Cefuroxime, Cefotetan, or other	1	1	0
3rd Generation Cephalosporin (e.g. Cefixime, Cefotaxime, Ceftriaxone, Ceftazidime)	7	60	0
4th Generation Cephalosporin (e.g. Cefipime)	6	51	10
Aminoglycosides: eg Gentamicin, Tobramycin, Amikacin	0	2	0
Amoxicillin, Ampicillin	48	17	2
Betalactam/Betalactamase inhibitor combination: eg Ampicillin-Clavunate, Piperacillin-Tazobactam, or other	2	0	0
Carbapenem (e.g. Imipenem, Meropenem, Ertapenem)	0	60	7
Chloramphenicol	0	0	2
Cotrimoxazole (trimethoprim-sulfamethoxazole)	41	21	5
Fluoroquinolone (e.g. Ciprofloxacin, Norfloxacin, Ofloxacin, Levofloxacin)	12	53	2
Macrolide (e.g. Azithromycin, Erythromycin, Clarithromycin)	0	0	2
Nitrofurantoin	0	0	2
Penicillin	2	0	0
Polymyxins (e.g. Colistin)	0	38	29
Tigecycline	0	2	0

© 2025 Amatya B et al. *JAMA Network Open*.

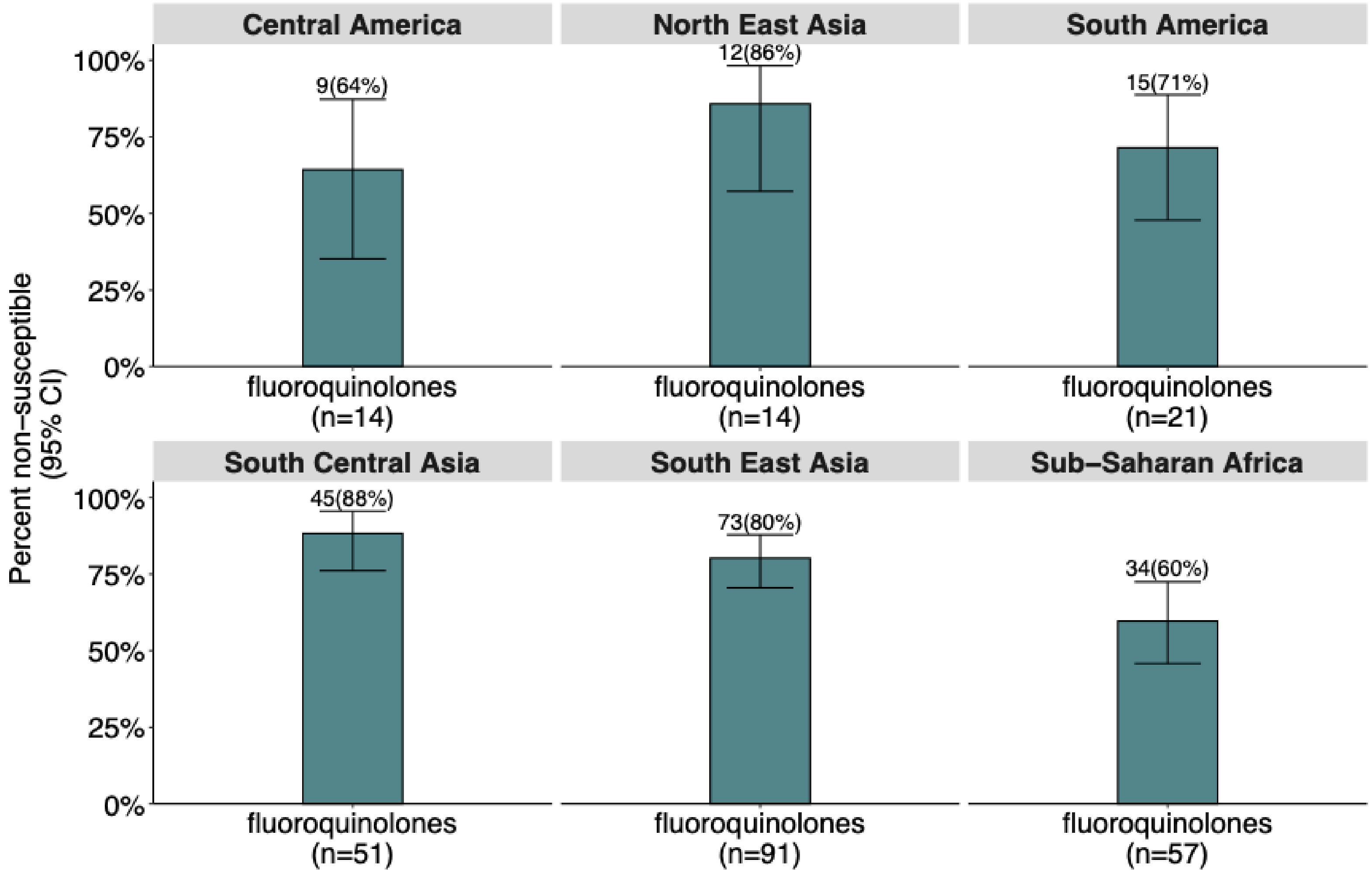

eFigure 10. Non-susceptibility pattern of *Campylobacter* species to fluoroquinolones stratified by world regions.

© 2025 Amatya B et al. *JAMA Network Open*.

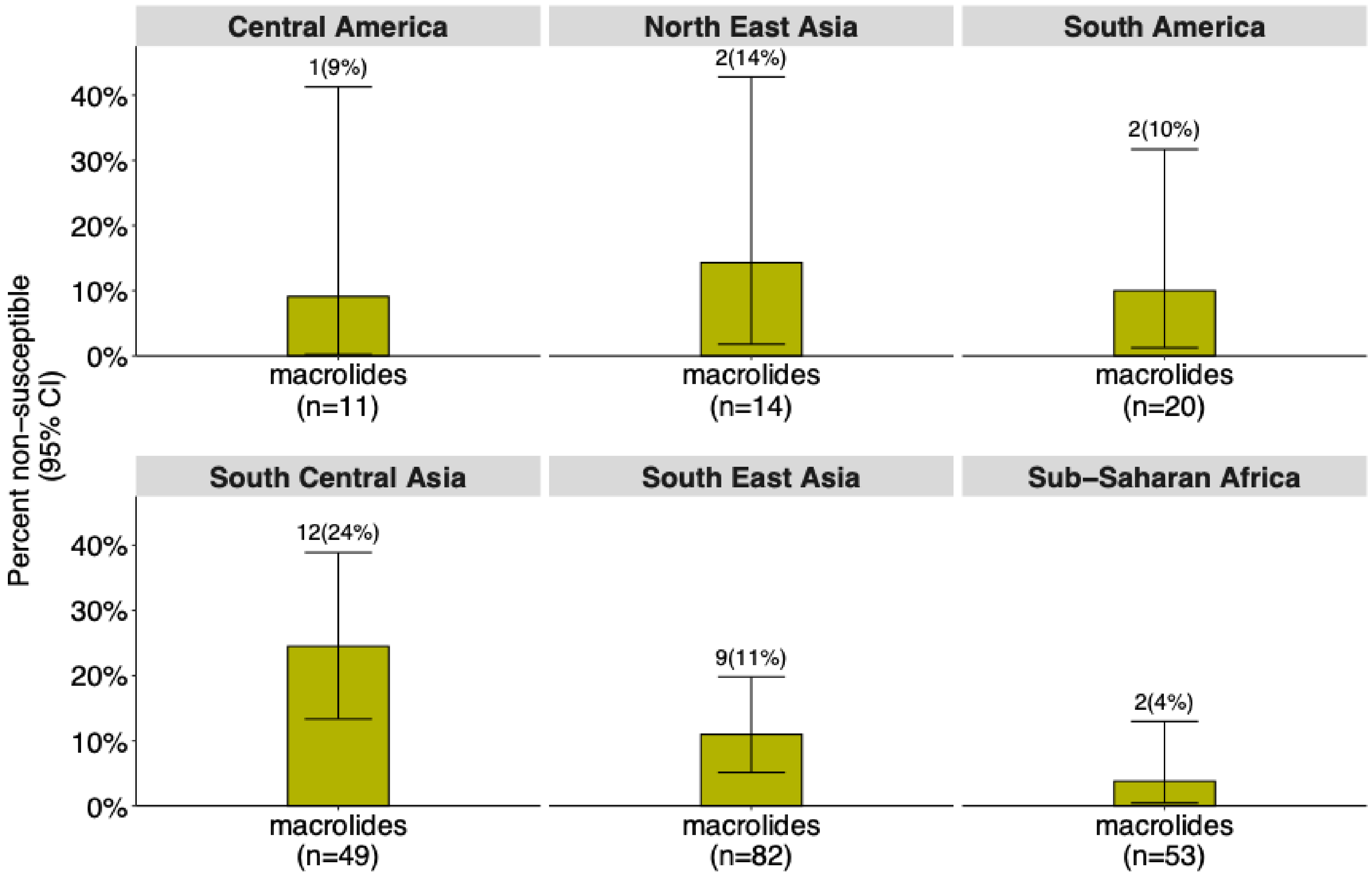

eFigure 11. Non-susceptibility pattern of *Campylobacter* species to macrolides stratified by world regions.

© 2025 Amatya B et al. *JAMA Network Open*.

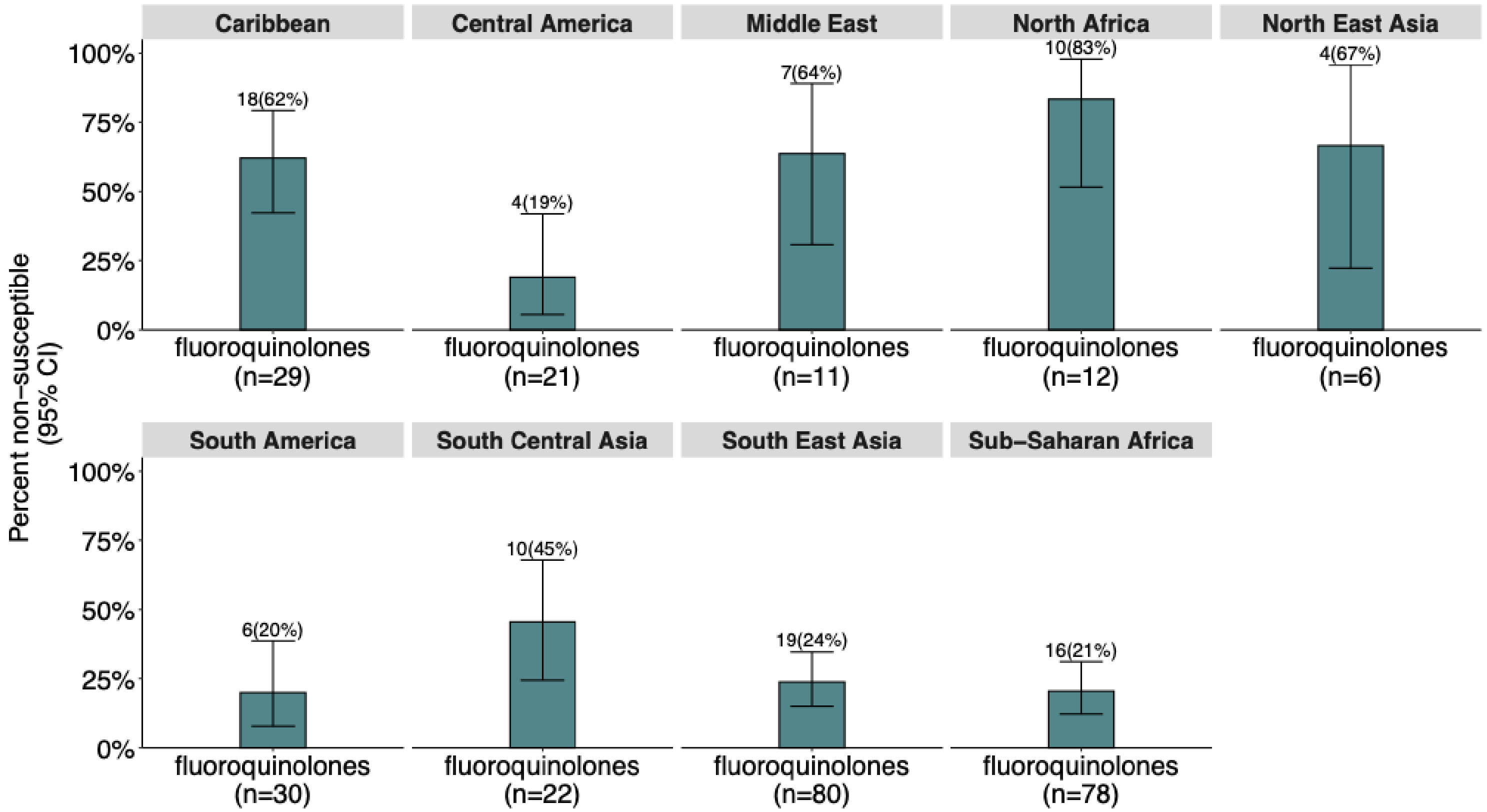

eFigure 12. Non-susceptibility pattern of non-typhoidal *Salmonella* species to fluoroquinolones stratified by world regions.

© 2025 Amatya B et al. *JAMA Network Open*.

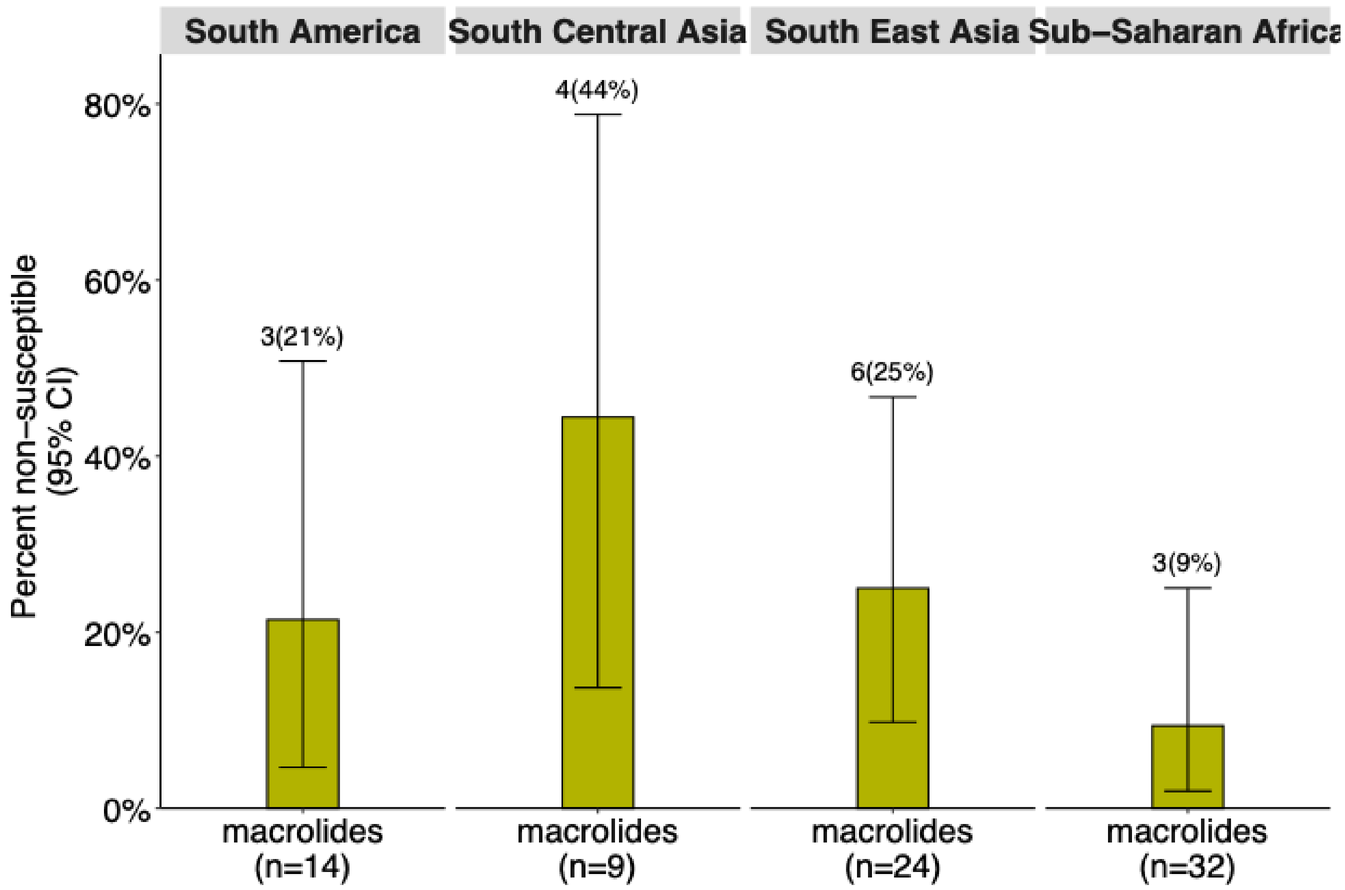

eFigure 13. Non-susceptibility pattern of non-typhoidal *Salmonella* species to macrolides stratified by world regions.

© 2025 Amatya B et al. *JAMA Network Open*.

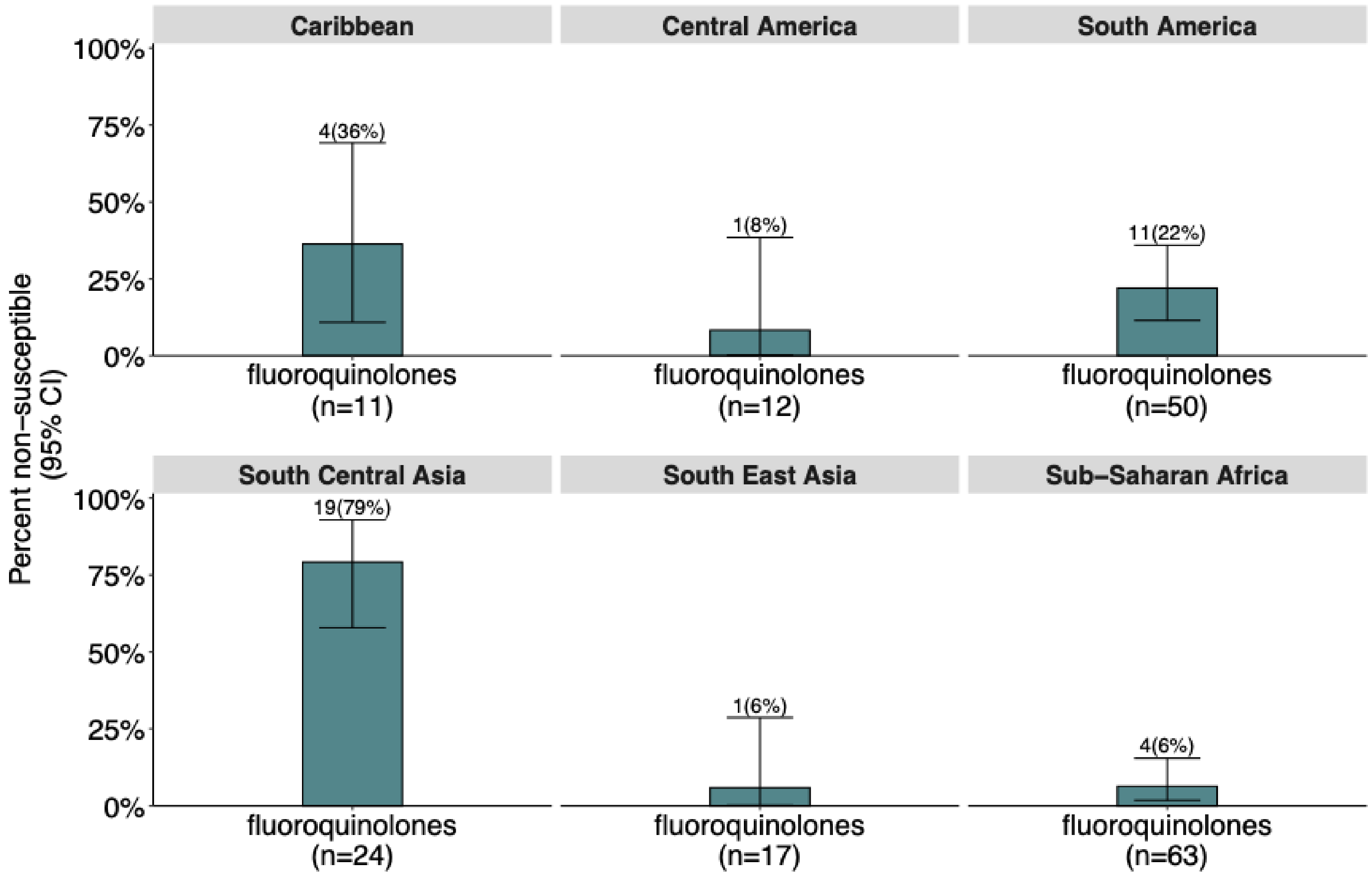

eFigure 14. Antibiotic susceptibility pattern of Shigella species to fluoroquinolones stratified by world regions.

© 2025 Amatya B et al. *JAMA Network Open*.

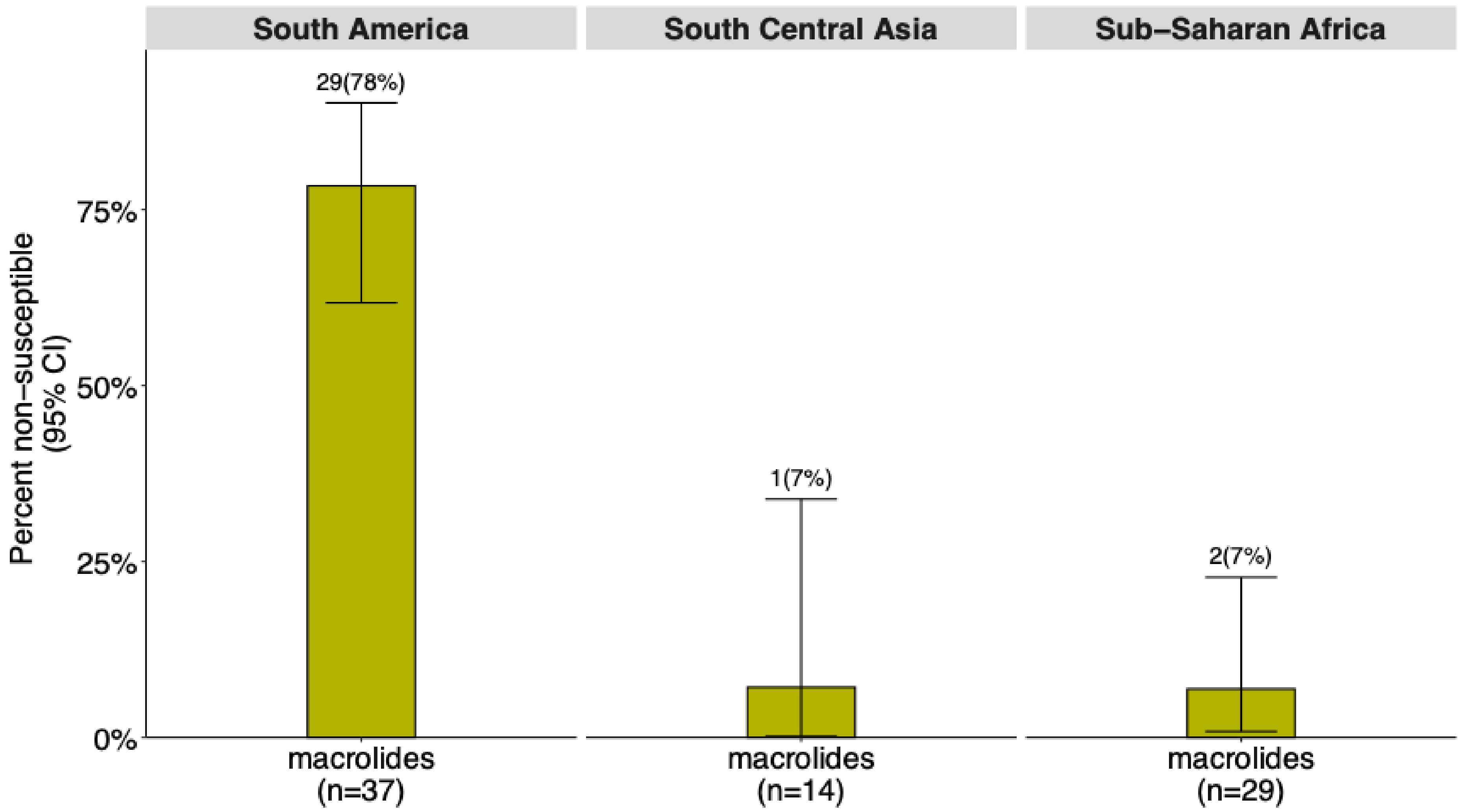

eFigure 15. Antibiotic susceptibility pattern of Shigella species to macrolides stratified by world regions.

© 2025 Amatya B et al. *JAMA Network Open*.

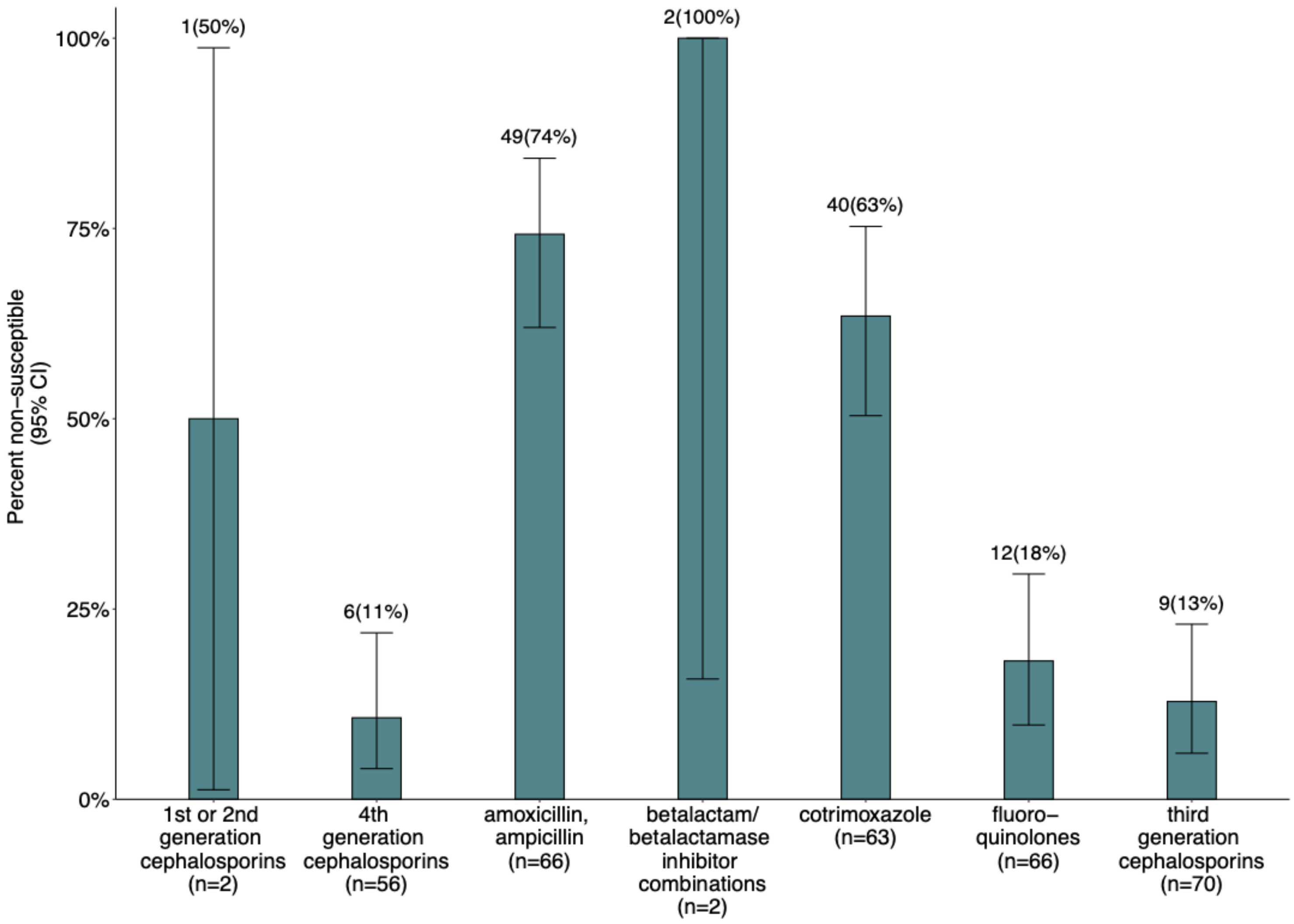

eFigure 16. Antibiotic susceptibility pattern of diarrheagenic *E. coli* to different antibiotics (n=75).

© 2025 Amatya B et al. *JAMA Network Open*.

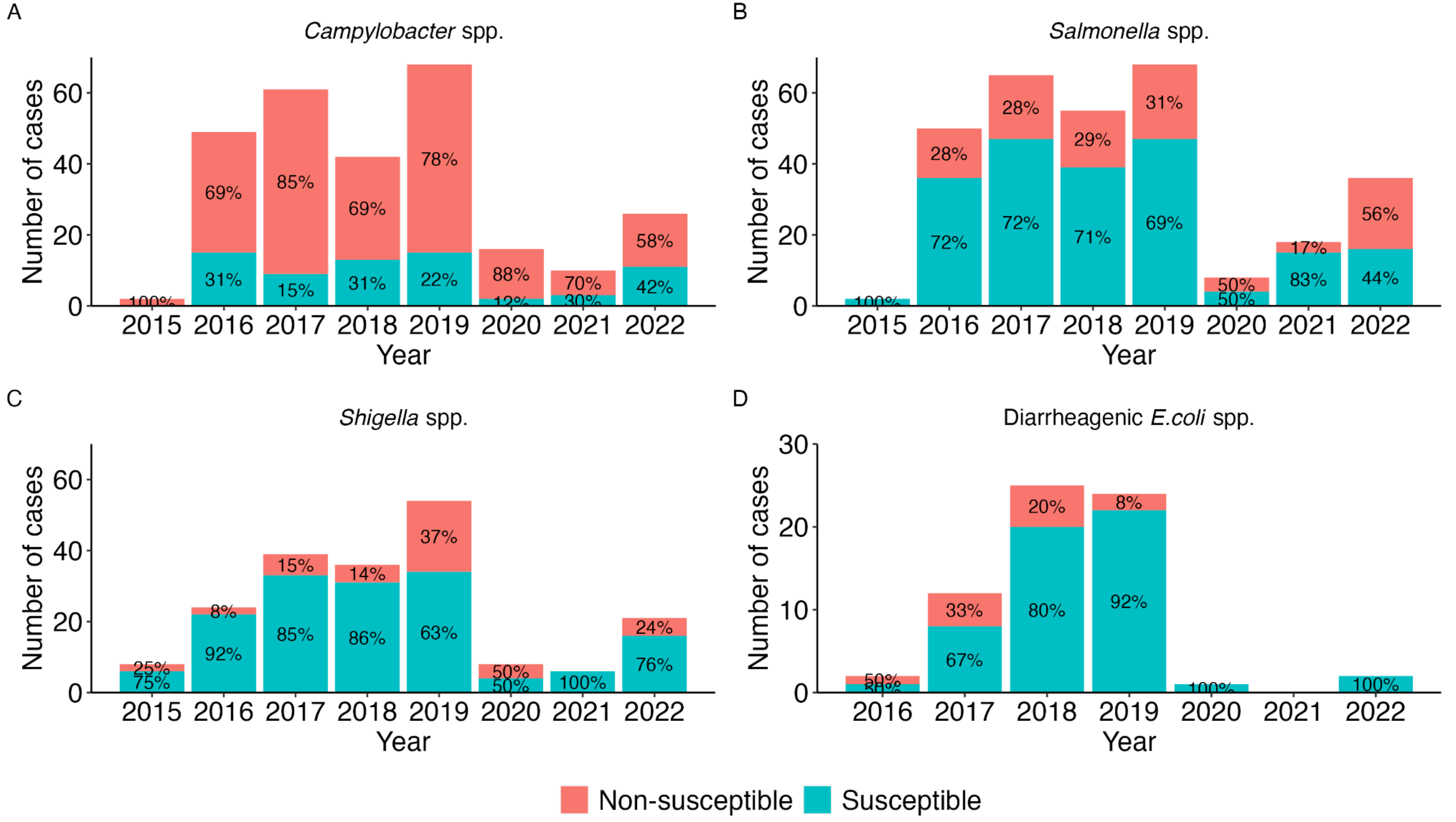

eFigure 17. Temporal trends of AST of fluoroquinolones from 2015-2022 by bacteria; A. *Campylobacter* species, B. Non-typhoidal *Salmonella* species, C. *Shigella* species and D. Diarrheagenic *E. coli* species.

© 2025 Amatya B et al. *JAMA Network Open*.

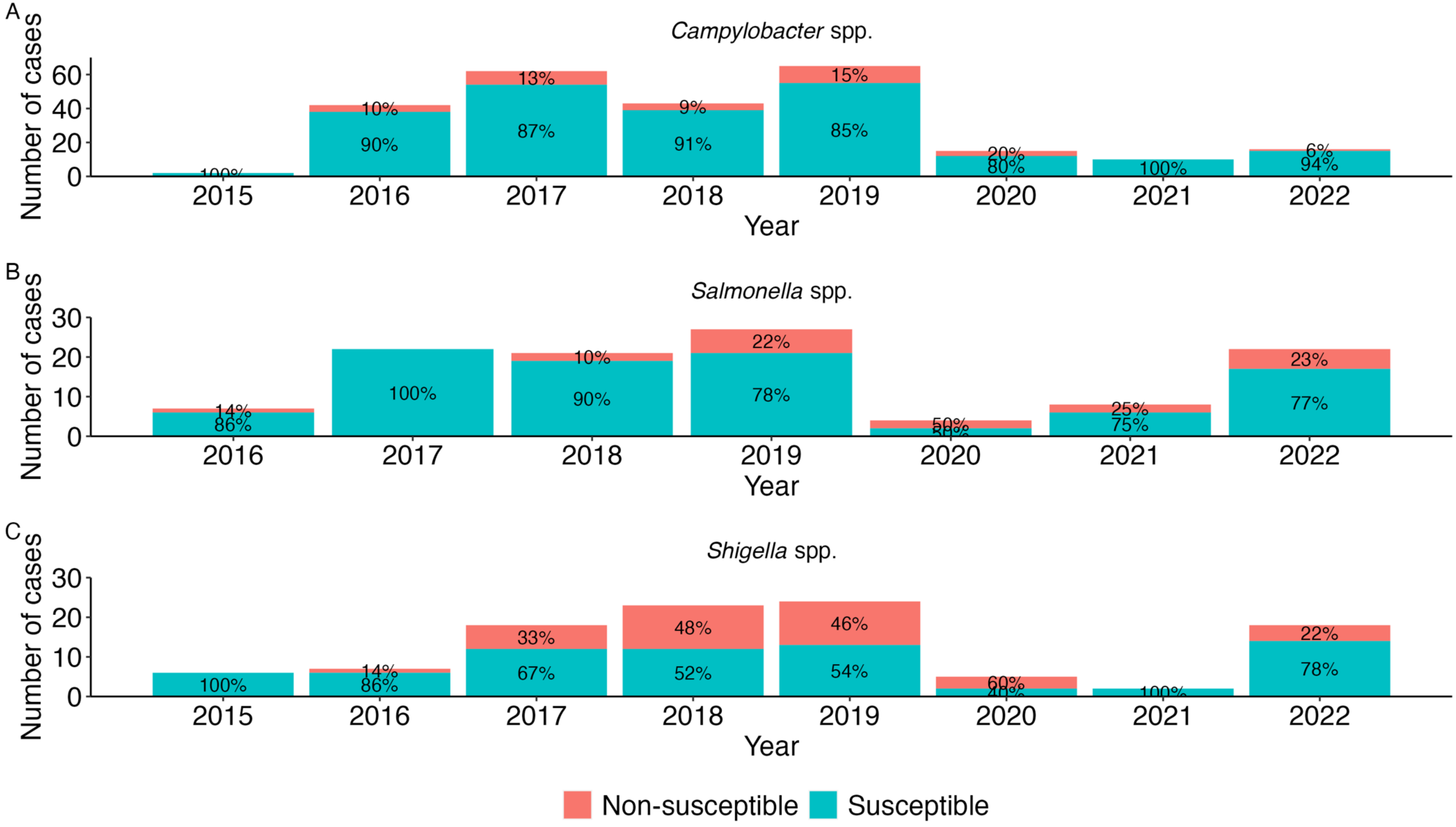

eFigure 18. Temporal trends of AST of macrolides from 2015-2022 by bacteria; A. *Campylobacter* species, B. Non-typhoidal *Salmonella* species, C. *Shigella* species.

© 2025 Amatya B et al. *JAMA Network Open*.

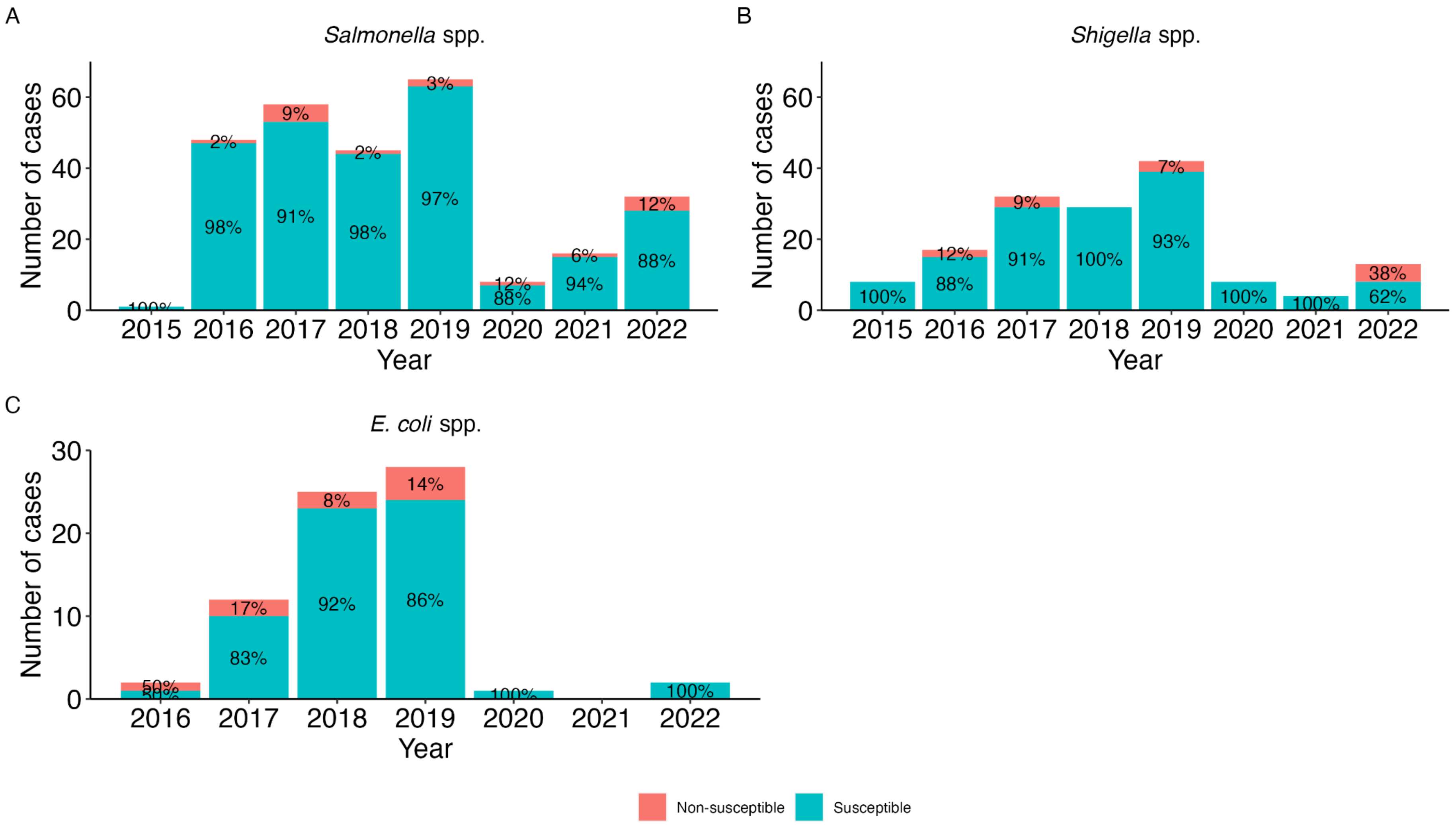

eFigure 19. Temporal trends of AST of third generation cephalosporins from 2015-2022 by bacteria; A. Non-typhoidal *Salmonella* species, B. *Shigella* species, C. diarrheagenic *E. coli* species.

© 2025 Amatya B et al. *JAMA Network Open*.

eTable 7. Table showing missing data patterns i.e. unknown/not done/not reported by organism, antibiotic class and region.

Organism	Antibiotic class	Region	Unknown/not done/not reported (n)
Campylobacter	Fluoroquinolone (e.g. Ciprofloxacin, Norfloxacin, Ofloxacin, Levofloxacin)	Caribbean	1
Campylobacter	Fluoroquinolone (e.g. Ciprofloxacin, Norfloxacin, Ofloxacin, Levofloxacin)	North America	1
Campylobacter	Fluoroquinolone (e.g. Ciprofloxacin, Norfloxacin, Ofloxacin, Levofloxacin)	South Central Asia	2
Campylobacter	Fluoroquinolone (e.g. Ciprofloxacin, Norfloxacin, Ofloxacin, Levofloxacin)	South East Asia	3
Campylobacter	Fluoroquinolone (e.g. Ciprofloxacin, Norfloxacin, Ofloxacin, Levofloxacin)	Sub-Saharan Africa	1
Campylobacter	Macrolide (e.g. Azithromycin, Erythromycin, Clarithromycin)	Central America	3
Campylobacter	Macrolide (e.g. Azithromycin, Erythromycin, Clarithromycin)	North Africa	2
Campylobacter	Macrolide (e.g. Azithromycin, Erythromycin, Clarithromycin)	South America	1
Campylobacter	Macrolide (e.g. Azithromycin, Erythromycin, Clarithromycin)	South Central Asia	4
Campylobacter	Macrolide (e.g. Azithromycin, Erythromycin, Clarithromycin)	South East Asia	12
Campylobacter	Macrolide (e.g. Azithromycin, Erythromycin, Clarithromycin)	Sub-Saharan Africa	5
E. Coli	4th Generation Cephalosporin (e.g. Cefipime)	Caribbean	2
E. Coli	4th Generation Cephalosporin (e.g. Cefipime)	North Africa	1
E. Coli	4th Generation Cephalosporin (e.g. Cefipime)	North East Asia	1
E. Coli	4th Generation Cephalosporin (e.g. Cefipime)	South East Asia	3
E. Coli	4th Generation Cephalosporin (e.g. Cefipime)	Sub-Saharan Africa	4
E. Coli	4th Generation Cephalosporin (e.g. Cefipime)	Western Europe	3
E. Coli	Amoxicillin, Ampicillin	South America	1

© 2025 Amatya B et al. *JAMA Network Open*.

E. Coli	Amoxicillin, Ampicillin	Sub-Saharan Africa	1
E. Coli	Amoxicillin, Ampicillin	Western Europe	2
E. Coli	Carbapenem (e.g. Imipenem, Meropenem, Ertapenem)	North East Asia	1
E. Coli	Carbapenem (e.g. Imipenem, Meropenem, Ertapenem)	South America	3
E. Coli	Carbapenem (e.g. Imipenem, Meropenem, Ertapenem)	Sub-Saharan Africa	2
E. Coli	Carbapenem (e.g. Imipenem, Meropenem, Ertapenem)	Western Europe	1
E. Coli	Chloramphenicol	South Central Asia	1
E. Coli	Chloramphenicol	South East Asia	1
E. Coli	Cotrimoxazole (trimethoprim-sulfamethoxazole)	North Africa	1
E. Coli	Cotrimoxazole (trimethoprim-sulfamethoxazole)	South America	1
E. Coli	Cotrimoxazole (trimethoprim-sulfamethoxazole)	South East Asia	1
E. Coli	Cotrimoxazole (trimethoprim-sulfamethoxazole)	Sub-Saharan Africa	3
E. Coli	Cotrimoxazole (trimethoprim-sulfamethoxazole)	Western Europe	1
E. Coli	Fluoroquinolone (e.g. Ciprofloxacin, Norfloxacin, Ofloxacin, Levofloxacin)	North Africa	1
E. Coli	Fluoroquinolone (e.g. Ciprofloxacin, Norfloxacin, Ofloxacin, Levofloxacin)	North East Asia	1
E. Coli	Fluoroquinolone (e.g. Ciprofloxacin, Norfloxacin, Ofloxacin, Levofloxacin)	Sub-Saharan Africa	2
E. Coli	Macrolide (e.g. Azithromycin, Erythromycin, Clarithromycin)	South Central Asia	1
E. Coli	Macrolide (e.g. Azithromycin, Erythromycin, Clarithromycin)	South East Asia	1
E. Coli	Nitrofurantoin	South Central Asia	1
E. Coli	Nitrofurantoin	South East Asia	1
E. Coli	Polymyxins (e.g. Colistin)	Caribbean	2
E. Coli	Polymyxins (e.g. Colistin)	Central America	2
E. Coli	Polymyxins (e.g. Colistin)	Middle East	2

© 2025 Amatya B et al. *JAMA Network Open*.

E. Coli	Polymyxins (e.g. Colistin)	North Africa	1
E. Coli	Polymyxins (e.g. Colistin)	North East Asia	1
E. Coli	Polymyxins (e.g. Colistin)	South America	10
E. Coli	Polymyxins (e.g. Colistin)	South Central Asia	1
E. Coli	Polymyxins (e.g. Colistin)	South East Asia	5
E. Coli	Polymyxins (e.g. Colistin)	Sub-Saharan Africa	5
E. Coli	Polymyxins (e.g. Colistin)	Western Europe	3
Salmonella Species	3rd Generation Cephalosporin (e.g. Cefixime, Cefotaxime, Ceftriaxone, Ceftazidime)	Caribbean	9
Salmonella Species	3rd Generation Cephalosporin (e.g. Cefixime, Cefotaxime, Ceftriaxone, Ceftazidime)	Central America	4
Salmonella Species	3rd Generation Cephalosporin (e.g. Cefixime, Cefotaxime, Ceftriaxone, Ceftazidime)	Middle East	2
Salmonella Species	3rd Generation Cephalosporin (e.g. Cefixime, Cefotaxime, Ceftriaxone, Ceftazidime)	North Africa	4
Salmonella Species	3rd Generation Cephalosporin (e.g. Cefixime, Cefotaxime, Ceftriaxone, Ceftazidime)	North East Asia	1
Salmonella Species	3rd Generation Cephalosporin (e.g. Cefixime, Cefotaxime, Ceftriaxone, Ceftazidime)	South America	5
Salmonella Species	3rd Generation Cephalosporin (e.g. Cefixime, Cefotaxime, Ceftriaxone, Ceftazidime)	South Central Asia	8
Salmonella Species	3rd Generation Cephalosporin (e.g. Cefixime, Cefotaxime, Ceftriaxone, Ceftazidime)	South East Asia	17
Salmonella Species	3rd Generation Cephalosporin (e.g. Cefixime, Cefotaxime, Ceftriaxone, Ceftazidime)	Sub-Saharan Africa	11
Salmonella Species	3rd Generation Cephalosporin (e.g. Cefixime, Cefotaxime, Ceftriaxone, Ceftazidime)	Western Europe	2
Salmonella Species	Amoxicillin, Ampicillin	Middle East	3

© 2025 Amatya B et al. *JAMA Network Open*.

Salmonella Species	Amoxicillin, Ampicillin	North Africa	2
Salmonella Species	Amoxicillin, Ampicillin	North America	1
Salmonella Species	Amoxicillin, Ampicillin	North East Asia	1
Salmonella Species	Amoxicillin, Ampicillin	South America	2
Salmonella Species	Amoxicillin, Ampicillin	South Central Asia	4
Salmonella Species	Amoxicillin, Ampicillin	South East Asia	8
Salmonella Species	Amoxicillin, Ampicillin	Sub-Saharan Africa	21
Salmonella Species	Amoxicillin, Ampicillin	Western Europe	2
Salmonella Species	Carbapenem (e.g. Imipenem, Meropenem, Ertapenem)	Caribbean	24
Salmonella Species	Carbapenem (e.g. Imipenem, Meropenem, Ertapenem)	Central America	14
Salmonella Species	Carbapenem (e.g. Imipenem, Meropenem, Ertapenem)	Middle East	7
Salmonella Species	Carbapenem (e.g. Imipenem, Meropenem, Ertapenem)	North Africa	6
Salmonella Species	Carbapenem (e.g. Imipenem, Meropenem, Ertapenem)	North East Asia	2
Salmonella Species	Carbapenem (e.g. Imipenem, Meropenem, Ertapenem)	Oceania	3
Salmonella Species	Carbapenem (e.g. Imipenem, Meropenem, Ertapenem)	South America	22
Salmonella Species	Carbapenem (e.g. Imipenem, Meropenem, Ertapenem)	South Central Asia	15

© 2025 Amatya B et al. *JAMA Network Open*.

Salmonella Species	Carbapenem (e.g. Imipenem, Meropenem, Ertapenem)	South East Asia	52
Salmonella Species	Carbapenem (e.g. Imipenem, Meropenem, Ertapenem)	Sub-Saharan Africa	50
Salmonella Species	Carbapenem (e.g. Imipenem, Meropenem, Ertapenem)	Western Europe	3
Salmonella Species	Cotrimoxazole (trimethoprim-sulfamethoxazole)	Caribbean	3
Salmonella Species	Cotrimoxazole (trimethoprim-sulfamethoxazole)	Central America	1
Salmonella Species	Cotrimoxazole (trimethoprim-sulfamethoxazole)	Middle East	1
Salmonella Species	Cotrimoxazole (trimethoprim-sulfamethoxazole)	North Africa	1
Salmonella Species	Cotrimoxazole (trimethoprim-sulfamethoxazole)	North East Asia	1
Salmonella Species	Cotrimoxazole (trimethoprim-sulfamethoxazole)	Oceania	1
Salmonella Species	Cotrimoxazole (trimethoprim-sulfamethoxazole)	South America	3
Salmonella Species	Cotrimoxazole (trimethoprim-sulfamethoxazole)	South Central Asia	7
Salmonella Species	Cotrimoxazole (trimethoprim-sulfamethoxazole)	South East Asia	26
Salmonella Species	Cotrimoxazole (trimethoprim-sulfamethoxazole)	Sub-Saharan Africa	14
Salmonella Species	Cotrimoxazole (trimethoprim-sulfamethoxazole)	Western Europe	1
Salmonella Species	Fluoroquinolone (e.g. Ciprofloxacin, Norfloxacin, Ofloxacin, Levofloxacin)	North Africa	2
Salmonella Species	Fluoroquinolone (e.g. Ciprofloxacin, Norfloxacin, Ofloxacin, Levofloxacin)	South America	3

© 2025 Amatya B et al. *JAMA Network Open*.

Salmonella Species	Fluoroquinolone (e.g. Ciprofloxacin, Norfloxacin, Ofloxacin, Levofloxacin)	South Central Asia	3
Salmonella Species	Fluoroquinolone (e.g. Ciprofloxacin, Norfloxacin, Ofloxacin, Levofloxacin)	South East Asia	12
Salmonella Species	Fluoroquinolone (e.g. Ciprofloxacin, Norfloxacin, Ofloxacin, Levofloxacin)	Sub-Saharan Africa	11
Salmonella Species	Macrolide (e.g. Azithromycin, Erythromycin, Clarithromycin)	Caribbean	24
Salmonella Species	Macrolide (e.g. Azithromycin, Erythromycin, Clarithromycin)	Central America	16
Salmonella Species	Macrolide (e.g. Azithromycin, Erythromycin, Clarithromycin)	Middle East	7
Salmonella Species	Macrolide (e.g. Azithromycin, Erythromycin, Clarithromycin)	North Africa	6
Salmonella Species	Macrolide (e.g. Azithromycin, Erythromycin, Clarithromycin)	North East Asia	3
Salmonella Species	Macrolide (e.g. Azithromycin, Erythromycin, Clarithromycin)	Oceania	4
Salmonella Species	Macrolide (e.g. Azithromycin, Erythromycin, Clarithromycin)	South America	19
Salmonella Species	Macrolide (e.g. Azithromycin, Erythromycin, Clarithromycin)	South Central Asia	16
Salmonella Species	Macrolide (e.g. Azithromycin, Erythromycin, Clarithromycin)	South East Asia	68
Salmonella Species	Macrolide (e.g. Azithromycin, Erythromycin, Clarithromycin)	Sub-Saharan Africa	57
Salmonella Species	Macrolide (e.g. Azithromycin, Erythromycin, Clarithromycin)	Western Europe	2
Shigella Species	3rd Generation Cephalosporin (e.g. Cefixime, Cefotaxime, Ceftriaxone, Ceftazidime)	Caribbean	7
Shigella Species	3rd Generation Cephalosporin (e.g. Cefixime, Cefotaxime, Ceftriaxone, Ceftazidime)	Central America	6

© 2025 Amatya B et al. *JAMA Network Open*.

Shigella Species	3rd Generation Cephalosporin (e.g. Cefixime, Cefotaxime, Ceftriaxone, Ceftazidime)	Middle East	1
Shigella Species	3rd Generation Cephalosporin (e.g. Cefixime, Cefotaxime, Ceftriaxone, Ceftazidime)	North Africa	4
Shigella Species	3rd Generation Cephalosporin (e.g. Cefixime, Cefotaxime, Ceftriaxone, Ceftazidime)	South America	6
Shigella Species	3rd Generation Cephalosporin (e.g. Cefixime, Cefotaxime, Ceftriaxone, Ceftazidime)	South Central Asia	4
Shigella Species	3rd Generation Cephalosporin (e.g. Cefixime, Cefotaxime, Ceftriaxone, Ceftazidime)	South East Asia	3
Shigella Species	3rd Generation Cephalosporin (e.g. Cefixime, Cefotaxime, Ceftriaxone, Ceftazidime)	Sub-Saharan Africa	22
Shigella Species	Amoxicillin, Ampicillin	North Africa	1
Shigella Species	Amoxicillin, Ampicillin	South America	2
Shigella Species	Amoxicillin, Ampicillin	South Central Asia	1
Shigella Species	Amoxicillin, Ampicillin	Sub-Saharan Africa	5
Shigella Species	Cotrimoxazole (trimethoprim-sulfamethoxazole)	Caribbean	1
Shigella Species	Cotrimoxazole (trimethoprim-sulfamethoxazole)	Central America	1
Shigella Species	Cotrimoxazole (trimethoprim-sulfamethoxazole)	Middle East	1
Shigella Species	Cotrimoxazole (trimethoprim-sulfamethoxazole)	North Africa	1
Shigella Species	Cotrimoxazole (trimethoprim-sulfamethoxazole)	South Central Asia	2
Shigella Species	Cotrimoxazole (trimethoprim-sulfamethoxazole)	South East Asia	2
Shigella Species	Cotrimoxazole (trimethoprim-sulfamethoxazole)	Sub-Saharan Africa	9
Shigella Species	Cotrimoxazole (trimethoprim-sulfamethoxazole)	Western Europe	1
Shigella Species	Fluoroquinolone (e.g. Ciprofloxacin, Norfloxacin, Ofloxacin, Levofloxacin)	South Central Asia	3

© 2025 Amatya B et al. *JAMA Network Open*.

Shigella Species	Fluoroquinolone (e.g. Ciprofloxacin, Norfloxacin, Ofloxacin, Levofloxacin)	Sub-Saharan Africa	8
Shigella Species	Macrolide (e.g. Azithromycin, Erythromycin, Clarithromycin)	Caribbean	7
Shigella Species	Macrolide (e.g. Azithromycin, Erythromycin, Clarithromycin)	Central America	9
Shigella Species	Macrolide (e.g. Azithromycin, Erythromycin, Clarithromycin)	Middle East	3
Shigella Species	Macrolide (e.g. Azithromycin, Erythromycin, Clarithromycin)	North Africa	5
Shigella Species	Macrolide (e.g. Azithromycin, Erythromycin, Clarithromycin)	South America	13
Shigella Species	Macrolide (e.g. Azithromycin, Erythromycin, Clarithromycin)	South Central Asia	12
Shigella Species	Macrolide (e.g. Azithromycin, Erythromycin, Clarithromycin)	South East Asia	10
Shigella Species	Macrolide (e.g. Azithromycin, Erythromycin, Clarithromycin)	Sub-Saharan Africa	42
Shigella Species	Macrolide (e.g. Azithromycin, Erythromycin, Clarithromycin)	Western Europe	2

© 2025 Amatya B et al. *JAMA Network Open*.

eTable 8. Diarrheagenic *E. coli* non-susceptibility excluding the Lima site

Antibiotic class	AST pattern	n	%
Amoxicillin, Ampicillin	Non-susceptible	13	54
Cotrimoxazole (trimethoprim-sulfamethoxazole)	Non-susceptible	7	33
3rd Generation Cephalosporin (e.g. Cefixime, Cefotaxime, Ceftriaxone, Ceftazidime)	Non-susceptible	6	22
4th Generation Cephalosporin (e.g. Cefipime)	Non-susceptible	3	23
Fluoroquinolone (e.g. Ciprofloxacin, Norfloxacin, Ofloxacin, Levofloxacin)	Non-susceptible	3	13
Betalactam/Betalactamase inhibitor combination: eg Ampicillin-Clavunate, Piperacillin-Tazobactam, or other	Non-susceptible	2	100
Penicillin	Non-susceptible	2	100
1st or 2nd Generation Cephalosporin: Cefazolin, Cephalexin, Cefadroxil, Cefoxitin, Cefuroxime, Cefotetan, or other	Non-susceptible	1	50

© 2025 Amatya B et al. *JAMA Network Open*.

eAppendix. Supplemental results and discussion

Analysis by world regions

Sub-Saharan Africa

Campylobacter species were non-susceptible to fluoroquinolones in 34/57 (60%; 95% CI, 0.46-0.72) cases (Figure 1A, eFigure 10) and to macrolides in 2/53 (4%, 95% CI, 0.00-0.13) of cases (Figure 1B, eFigure 11). NTS were non-susceptible to fluoroquinolones in 16/78 (21%; 95% CI, 0.12-0.31) of cases (Figure 2A, eFigure 12), and to macrolides in 3/32 (9%; 95% CI, 0.02-0.25) (Figure 2B, eFigure 13). *Shigella* species were non-susceptible to fluoroquinolones in 4/63 (6%; 95% CI, 0.02-0.15) (Figure 3A, eFigure 14), and to macrolides in 2/29 (7%, 95% CI, 0.01-0.23) (Figure 3B, eFigure 15). Among limited isolates (n=6), there was no reported non-susceptibility to fluoroquinolones among diarrheagenic *E. coli* in this region.

© 2025 Amatya B et al. *JAMA Network Open*.

Southeast Asia

Campylobacter species were non-susceptible to fluoroquinolones in 73/91 (80%, 95% CI, 0.71-0.88) cases (Figure 1A), and to macrolides in 9/82 (11%) cases (Figure 1B). NTS were non-susceptible to fluoroquinolones in 19/80 (24%) (Figure 2A), and to macrolides in 6/24 (25%) cases (Figure 2B). *Shigella* species were non-susceptible to fluoroquinolones in 1/17 (6%) cases (Figure 3A), and to macrolides in 1/7 (14%) cases (Figure 3B). Among limited isolates (n=6), there was no reported non-susceptibility to fluoroquinolones.

South America

Campylobacter species were non-susceptible to fluoroquinolones in 15/21 (71%, 95% CI, 0.48-0.89) cases, and to macrolides in 2/20 (10%, 95% CI, 0.01-0.32) cases (Figure 1). NTS were non-susceptible to fluoroquinolones in 6/30 (20%/ 95% CI, 0.08-0.39) cases, and to macrolides in 3/14 (21%, 95% CI, 0.05-0.51) cases (Figure 2). *Shigella* species were non-susceptible to fluoroquinolones in 11/50 (22%; 95% CI, 0.12-0.36) cases, and to macrolides in 29/37 (78%; 95% CI, 0.62-0.90) cases (Figure 3). Diarrheagenic *E. coli* were non-susceptible to fluoroquinolones in 9/43 (21%; 95% CI, 0.10-0.36) cases (eFigure 16, Figure 4). Among macrolide non-susceptible Shigella cases from South America, 29 were reported from the Lima site, and 1 from the Spain site, i.e. testing was done at these sites. Most macrolide non-susceptible cases from South America were residents of Spain (6), but male and females were almost equal: 15 females and 14 males. *Shigella sonnei* macrolide non-susceptibility was equal among males and females in South America (12 each in those with type of Shigella data available). There was no data if

© 2025 Amatya B et al. *JAMA Network Open*.

the males were MSM as this information was not collected. Majority were travelers from Spain followed by USA. Among Shigella species from South America, macrolide testing was unknown/not done/not detected in 13/50 cases (not 71 total). There are no specified azithromycin breakpoints in CLSI or EUCAST hence, there may be site specific results.

South Central Asia

Campylobacter species were non-susceptible to fluoroquinolones in 45/51 (88%; 95% CI, 0.76-0.96), and to macrolides in 12/49 (24%; 95% CI, 0.13-0.39) (Figure 1). NTS were non-susceptible to fluoroquinolones in 10/22 (45%; 95% CI, 0.24-0.68), and to macrolides in 4/9 (44%; 95% CI, 0.14-0.79) (Figure 2). *Shigella* species were non-susceptible to fluoroquinolones in 19/24 (79%; 95% CI, 0.58-0.93), and to macrolides in 1/14 (7%; 95% CI, 0.00-0.34) (Figure 3). A single isolate of diarrheagenic *E. coli* (1/1, 100%; 95% CI, 0.03-1.00) was non-susceptible to fluoroquinolones (eFigure 16, Figure 4)

The Caribbean

Campylobacter species were non-susceptible to fluoroquinolones in 4/5 (80%; 95% CI, 0.28-0.99) cases, and to macrolides in 1/6 (17%; 95% CI, 0.00-0.64) cases (Figure 1). NTS were non-susceptible to fluoroquinolones in 18/29 (62%; 95% CI, 0.42-0.79) cases and no case of non-susceptibility to macrolides was reported from the Caribbean (Figure 2). *Shigella* species were non-susceptible to fluoroquinolones in 4/11 (36%;

© 2025 Amatya B et al. *JAMA Network Open*.

95% CI, 0.11-0.69) cases, and to macrolides in 1/4 (25%; 95% CI, 0.01-0.81) cases (Figure 3). Diarrheagenic *E. coli* were non-susceptible to fluoroquinolones in one of two cases from this region (95% CI, 0.01-0.99) (Figure 4).

Temporal trends

Looking at the temporal trends, for *Campylobacter*, fluoroquinolones non-susceptibility was the highest in 2017 at 85% (52/61). Macrolides non-susceptibility was the highest in 2019 at 15% (15/65).

For non-typhoidal *Salmonella*, fluoroquinolones non-susceptibility was the highest in 2022 at 56% (20/36). Macrolides non-susceptibility was the highest in 2022 at 23% (5/22). Third generation cephalosporins non-susceptibility was the highest in 2022 at 12% (4/32).

For *Shigella*, fluoroquinolones non-susceptibility was the highest in 2019 at 37% (20/54). Macrolide non-susceptibility was the highest (48%, 11/23) in 2018. For third generation cephalosporins, non-susceptibility was the highest in 2022 at 38% (5/13).

For diarrheagenic *E. coli*, fluoroquinolones non-susceptibility was the highest in 2017 at 33% (4/12). Third generation cephalosporins non-susceptibility was the highest in 2017 at 17% (2/12) (eFigures 17, 18, 19).

Visiting Friends and Relatives (VFRs)

© 2025 Amatya B et al. *JAMA Network Open*.

Most VFRs included in this study acquired diarrhea in Sub-Saharan Africa (27, 31%), followed by South Central Asia (20, 23%) and then South America (10, 11%). Among VFRs, fluoroquinolone non-susceptibility was 37% in *Campylobacter*, 8% in NTS, 7% in *Shigella*, 9% in fluoroquinolones. For macrolides in VFRs, macrolide non-susceptibility was 11% in *Campylobacter*, 2% in NTS, 3% in *Shigella*. These non-susceptibilities were less than that compared to all travelers.

© 2025 Amatya B et al. *JAMA Network Open*.
