# Supplementary material for: GeoSentinel Analysis of Travelers’ Diarrhea Antimicrobial Resistance Patterns
Source: JAMA Netw Open. 2025 Dec 22;8(12):e2551089. doi: 10.1001/jamanetworkopen.2025.51089 (PMC12723550; doi:10.1001/jamanetworkopen.2025.51089)
Supplement: Supplement 2. — Nonauthor Collaborators. GeoSentinel Surveillance Network Collaborators [file jamanetwopen-e2551089-s002.pdf]

Supplemental Online Content: Nonauthor Collaborators
*First name, last name, and suffix (if applicable) are required and will appear in PubMed.

*Group Name(s): GeoSentinel Surveillance Network							
***First Name and Middle Initial(s)**	***Last Name**	***Suffix (eg, Jr, III)**	Academic Degrees	Institution	Location (city, state/province, country)	Role or Contribution, eg, chair, principal investigator	Group (if more than 1 Group listed in the byline) and/or Subgroup (eg, Steering Committee)
Carsten Schade	Larsen						
Christian	Wejse						
Emmanuel	Bottieau						
Patrick	Soentjens						
Henry	Wu						
Noreen A.	Hynes						
Watcharapong	Piyaphanee						
Udomsak	Silachamroon						
Israel	Molina						
Fernando	Salvador						
Frank	Mockenhaupt						
Gundel Harms	Zwingenberger						
Alexandre	Duvignaud						
Denis	Malvy						
Francesco	Castelli						
Alberto	Matteeli						
Paul	Kelly						
Cosmina	Zeana						
Corneliu Petru	Popescu						
Susan	Kuhn						
Lin	Chen						
Marc	Mendelson						
Salim	Parker						
Félix	Djossou						
Cecilia	Perret						
Thomas	Weitzel						
Francois	Chappuis						
Matteo	Bassetti						
Sabine	Jordan						

Supplemental Online Content: Nonauthor Collaborators
*First name, last name, and suffix (if applicable) are required and will appear in PubMed.

***First Name and Middle Initial(s)**	***Last Name**	***Suffix (eg, Jr, III)**	Academic Degrees	Institution	Location (city, state/province, country)	Role or Contribution, eg, chair, principal investigator	Group (if more than 1 Group listed in the byline) and/or Subgroup (eg, Steering Committee)
Christof	Vinnemeier						
Jasper	Chan						
Kelvin	Chiu						
Eli	Schwartz						
Tamar	Lachish						
Christina	Greenaway						
Mauro	Saio						
Hugo	Siu						
Michael	Beadsworth						
Jose Antonio Perez	Molina						
Emilie	Javelle						
Sapha	Bakarati						
Cedric	Yansouni						
Arpita	Chakravarti						
Camilla	Rothe						
Mirjam	Schunk						
Andrea	Rossanese						
Ben	Wyler						
Paul Henri	Consigny						
Oula	Itani						
Terri	Sofarelli						
Ann	Settgast						
Hilmir	Ásgeirsson						
Mugen	Ujiie						
Kei	Yamamoto						
Shaun K.	Morris						
Katherine	Plewes						
Yazdan	Mirzanejad						
Pierre	Plourde						
Yukihiro	Yoshimura						

Supplemental Online Content: Nonauthor Collaborators
*First name, last name, and suffix (if applicable) are required and will appear in PubMed.

***First Name and Middle Initial(s)**	***Last Name**	***Suffix (eg, Jr, III)**	Academic Degrees	Institution	Location (city, state/province, country)	Role or Contribution, eg, chair, principal investigator	Group (if more than 1 Group listed in the byline) and/or Subgroup (eg, Steering Committee)
Natsuo	Tachikawa						
Patricia	Schlagenhauf						
Annelies	Zinkernage						
