# Supplementary material for: GeoSentinel Analysis of Travelers’ Diarrhea Antimicrobial Resistance Patterns
Source: JAMA Netw Open. 2025 Dec 22;8(12):e2551089. doi: 10.1001/jamanetworkopen.2025.51089 (PMC12723550; doi:10.1001/jamanetworkopen.2025.51089)
Supplement: Supplement 3. — Data Sharing Statement [file jamanetwopen-e2551089-s003.pdf]

Data Sharing Statement

Amatya. GeoSentinel Analysis of Travelers' Diarrhea Antimicrobial Resistance Patterns. *JAMA Netw Open*. Published December 22, 2025. doi:10.1001/jamanetworkopen.2025.51089

Data
Data available: No
